# Pan-Cancer Analysis of Enhancer-Induced PAN3-AS1 and Experimental Validation as a WFDC13-Promoting Factor in Colon Cancer

**DOI:** 10.32604/or.2025.069274

**Published:** 2025-12-30

**Authors:** Xu Guo, Yanan Yu, Xiaolin Ma, Yuanjie Cai

**Affiliations:** 1 Department of Breast Surgery, Zhejiang Hospital, Hangzhou, 310013, China; 2 Clinical Research Center, Affiliated Hospital of Shandong Second Medical University, Weifang, 261041, China; 3 Department of Oncology, Affiliated Hospital of Shandong Second Medical University, Weifang, 261041, China

**Keywords:** PAN3-AS1, pan-cancer, biomarker, immunotherapy, enhancer

## Abstract

**Background:**

Long non-coding RNAs (lncRNAs) act as epigenetic regulators for tumor hallmarks. This investigation sought to probe the carcinogenic trait of PAN3-AS1 across pan-cancer comprehensively.

**Methods:**

We studied the diagnostic and prognostic features and the immune landscape of PAN3-AS1 across pan-cancer by bioinformatics approaches. The hierarchical regulatory networks governing PAN3-AS1 expression in colon cancer were explored via chromatin immunoprecipitation, luciferase activity assays, and RNA immunoprecipitation, etc. We screened drugs sensitive to WAP four-disulfide core domain 13 (WFDC13) by virtual screening and molecular docking.

**Results:**

Single-cell transcriptomics demonstrated that a variety of immune populations abnormally expressed PAN3-AS1 beyond tumor cells. Integration of data from multiple databases revealed that PAN3-AS1 was highly expressed and associated with a bad prognosis in various malignancies. Notably, PAN3-AS1 expression was correlated with a suppressive immune microenvironment. Moreover, we observed poor immunotherapy efficacy when PAN3-AS1 was highly expressed in melanoma. *In vitro* assays and functional enrichment analysis revealed that PAN3-AS1 was associated with cell proliferation and the immune response in colon cancer. Our experiments confirmed that PAN3-AS1 facilitated WFDC13 expression through competitive binding to hsa-miR-423-5p in colon cancer. Moreover, the present paper illustrated that enhancer activity exerts an important modulatory ability for PAN3-AS1 expression.

**Conclusion:**

In short, PAN3-AS1 is a valuable biomarker for diagnosis and prognosis. PAN3-AS1 exhibits linkage to a cold tumor immune microenvironment (TME) and forecasts durable benefit from immunotherapy. Addressing the PAN3-AS1/miR-423-5p/WFDC13 axis might provide a novel option for improving immunotherapy efficacy in colon cancer.

## 1 Introduction

Based on the latest report released by the American Cancer Society, neoplasm remains a leading contributor to global mortality [1]. Traditional treatments such as surgery combined with chemotherapy have great limitations in clinical application due to drug resistance and severe side effects [2,3]. Currently, immunotherapy has made significant advancements in cancer management. Some molecular biomarkers, to name a few-microsatellite instability (MSI) [4], tumor mutational burden (TMB) [5], and programmed cell death 1/programmed cell death 1 ligand 1 (PD-1/PD-L1) [6], have grown into indispensable predictors of immunotherapy efficacy. If the tumor immune microenvironment (TIME) has low immune infiltration of T lymphocytes and abundant expression of immunosuppressive markers like PD-1/PD-L1, it is considered a “cold” tumor, which usually responds poorly to immunotherapy. Nevertheless, owing to the limitations of these current biomarkers, many patients cannot benefit from immunotherapy. Therefore, finding new biomarkers or therapeutic targets is an urgent need for immunotherapy.

Long chain non-coding RNAs (lncRNAs), that do not encode proteins, are typically more than 200 nucleotides in length [7]. In recent years, following the advancement of sequencing technologies, many key databases have been generated, among which are The Cancer Genome Atlas (TCGA) and The Genotype-Tissue Expression (GTEx). LncRNAs are widely acknowledged as important modulators in numerous diseases, notably tumors [8,9]. For instance, elevated expression of KCNQ1OT1 is prevalent in diverse cancers and is associated with adverse prognosis [10,11]. Furthermore, recent studies have shown that KCNQ1OT1 facilitates melanoma immune evasion through the miR-34a/STAT3/PD-L1 axis, indicating that KCNQ1OT1 is a potential immunotherapy target [12]. PAN3-AS1 is an antisense lncRNA from the PAN3 gene locus. The latest study has indicated that PAN3-AS1 may represent a promising biomarker for multiple sclerosis diagnosis [13]. However, current investigations into PAN3-AS1’s trait in cancer remain understudied and are limited to a single cancer type, pancreatic cancer. Ping et al. elucidated PAN3-AS1 as a ferroptosis-associated lncRNA that is correlated with the immune landscape [14]. Cao et al. identified PAN3-AS1 as a tumor angiogenesis-related lncRNA and validated its prognostic value [15].

This investigation sought to examine pan-cancer alterations of PAN3-AS1, encompassing its expression patterns, prognostic utility, and immune-related characteristics through bioinformatics strategies. Moreover, we wondered about the multi-level regulatory cascades controlling PAN3-AS1 overexpression in colon malignancy. Finally, we wanted to screen the sensitive drugs for the WAP four-disulfide core domain 13 (WFDC13) protein.

## 2 Materials and Methods

### 2.1 Gene Expression Signature and Survival Significance Analysis in Pan-Cancer

#### 2.1.1 Gene Expression Signature Analysis of PAN3-AS1

We first used the Sangerbox 3.0 database (http://sangerbox.com/ (accessed on 01 June 2025)) [16] to assess the tissue-specific expression patterns of PAN3-AS1 in 33 tumor types. The TCGA cohorts (https://www.cancer.gov/ccg/research/genome-sequencing/tcga (accessed on 01 June 2025)) were statistically characterized through the Assistant for Clinical Bioinformatics (ACLBI) database (https://www.aclbi.com (accessed on 01 June 2025)). In addition, we further quantified the expression divergence between neoplastic and adjacent non-tumor tissues via the University of Alabama at Birmingham Cancer Data Analysis Portal (UALCAN) database (http://ualcan.path.uab.edu/ (accessed on 01 June 2025)) [17].

#### 2.1.2 Single-Cell Sequencing Analysis of PAN3-AS1

The Tumor Immune Single-cell Hub 2 (TISCH2) database (http://tisch.comp-genomics.org/ (accessed on 01 June 2025)) [18] provides cell type annotations for a variety of tumor types by using the single-cell transcriptomics data. We acquired the following single-cell transcriptomics datasets (acute myeloid leukemia (AML)-GSE116256, basal cell carcinoma (BCC)-GSE123813, colorectal cancer (CRC)-GSE139555, head and neck squamous cell carcinoma (HNSC)-GSE103322, non-small cell lung cancer (NSCLC)-GSE127465, pancreatic adenocarcinoma (PAAD)-CRA001160) and quantified the expression divergence of PAN3-AS1 in single cells by generating fiddle plots.

#### 2.1.3 Profiling the Diagnostic and Prognostic Significance of PAN3-AS1

Gene Expression Profiling Interactive Analysis 2 (GEPIA2) [19] serves as a commonly used online tool (http://gepia2.cancer-pku.cn/ (accessed on 01 June 2025)). We analyzed the relevance between PAN3-AS1 expression in pan-cancer and clinical stage through the “stage plot” module. In addition, “stage plot” was selected in the “RNA-seq Web Tools” module of the lnc2Cancer3.0 database (http://bio-bigdata.hrbmu.edu.cn/lnc2cancer/ (accessed on 01 June 2025)) [20] to calculate the weight between PAN3-AS1 expression and pathological stages in diverse tumor types. Next, we used the Sangerbox 3.0 database to analyze the covariation of PAN3-AS1 with overall survival (OS), progression-free interval (PFI), disease-free interval (DFI), and disease-specific survival (DSS) in 44 malignancies. Outcome data are presented as Kaplan-Meier Plotter curves.

### 2.2 Immune Correlation Analysis across Pan-Cancer

#### 2.2.1 Linkage Analysis of PAN3-AS1 to the Immune Microenvironment

First, based on the pan-cancer datasets in the TCGA, the Pearson correlation coefficient linking PAN3-AS1 abundance and immune infiltration scores (immune score and estimate score) was analyzed for 44 cancers in the “Immune Infiltration” module using the Sangerbox 3.0 database. The data were converted by “TCGA” and log_2_(x + 0.001) to obtain. x indicates the expression level of genes. We subsequently calculated the correlation coefficient linking PAN3-AS1 abundance and immune-infiltrating cells using the XCELL algorithm through the ACLBI database. Furthermore, the linkage between the abundance of PAN3-AS1 and the immune checkpoints was analyzed using this platform, with a *p-*cutoff of 0.05.

#### 2.2.2 Prediction of Immunotherapy Efficacy by PAN3-AS1

We first used the Sangerbox 3.0 database to analyze the correspondence between the PAN3-AS1 abundance and mutant-allele tumor heterogeneity (MATH), TMB, MSI, and neoantigen (NEO) across pan-cancer. Next, the OS of melanoma patients undergoing PD-1 and CTLA4 antibodies was validated by “Ref_Therapy” in the “Query Gene” module of the Tumor Immune Dysfunction and Exclusion (TIDE) database (http://tide.dfci.harvard.edu/ (accessed on 01 June 2025)) [21]. Next, through the “Expression” panel, we analyzed the correlation of PAN3-AS1 with cytotoxic T cell (CTL).

#### 2.2.3 Linkage Analysis of WFDC13 with the Immune Microenvironment in Colon Adenocarcinoma (COAD)

First, the correlation coefficient linking WFDC13 abundance to immune cell infiltration types in COAD was determined in the Gene Set Cancer Analysis (GSCA) database (https://guolab.wchscu.cn/GSCA/#/ (accessed on 01 June 2025)). Additionally, the correlation coefficient linking WFDC13 abundance and immune cell infiltration, immune checkpoint abundance, TMB, NEO, and MANTIS score in COAD were analyzed by the Comprehensive Analysis on Multi-Omics of Immunotherapy in Pan-cancer (CAMOIP) database (https://www.camoip.net/ (accessed on 01 June 2025)). The MANTIS score was performed using MANTIS (version 1.0.3), an MSI-calling software.

#### 2.2.4 Gene Ontology (GO) and Kyoto Encyclopedia of Genes and Genomes (KEGG) Enrichment Analysis

We obtained RNA sequencing transcriptomic profiles of TCGA-COAD patients and analyzed the count data by the R package “DESeq2” (v1.36.0). We conducted median-based expression clustering of PAN3-AS1 among TCGA-COAD patients. The differentially expressed genes (DEGs) were extracted by log_2_(fold change) ≥ 1 and *p* < 0.05. Volcano maps of DEGs were plotted via the R packages ‘ggpubr’ (v0.4.0) and ‘ggthemes’ (v5.1.0). Heatmaps of the DEGs were plotted through the R package ‘pheatmap’ (v1.0.12). GO/KEGG analysis can predict the biological feature of target genes with reference to the overall abundance of the transcriptome [22]. Next, GO/KEGG enrichment findings were visualized as bubble plots via the Sangerbox 3.0 database.

### 2.3 Molecular Assays

#### 2.3.1 Cell Culture

This experiment was conducted using the human colon cancer cell lines HCT116, HT29, DLD1, and SW480, human 293T cells, and the normal colon epithelial cell line FHC. The cells utilised in this paper were derived from the Cell Bank of the Chinese Academy of Sciences (Shanghai, China). HCT116 and HT29 cells were cultured at 37°C and in a 5% CO_2_ cell incubator in McCoy’s 5A medium (Gibco, 16600108, Thermo Fisher Scientific, Waltham, MA, USA) containing 10% fetal bovine serum (Gibco, 10100147C, Thermo Fisher Scientific). DLD1 cells were cultured at 37°C and in a 5% CO_2_ cell incubator in RPMI 1640 medium (Gibco, 11875093, Thermo Fisher Scientific) containing 10% fetal bovine serum. SW480 cells were cultured at 37°C without CO_2_ in L15 medium (Gibco, 11415064, Thermo Fisher Scientific) containing 10% fetal bovine serum. 293T and FHC cells were cultured at 37°C and in a 5% CO_2_ cell incubator in DMEM medium (Gibco, 10566024, Thermo Fisher Scientific) containing 10% fetal bovine serum. Mycoplasma screening was conducted bimonthly on all cell lines, with short tandem repeat (STR) profiling for authentication.

#### 2.3.2 siRNA Transfection

One day before transfection, cells were distributed into 6-well plates at a density of 1 × 10^5^ cells/mL. Two hours before transfection, the culture medium was converted to serum-free and antibiotic-free to reduce the effects of serum and antibiotics on the transfection efficiency. We used Lipofectamine™ RNAiMAX (Invitrogen, 13778150, Carlsbad, CA, USA) as the transfection vehicle. The siRNAs were derived from GenePharma (Shanghai, China). The final concentration of siRNA was 10 nM. The total time for transfection was 72 h. The siRNA sequences employed in this study are provided below:

PAN3-AS1-siRNA-1# S: GGCUCUACCUGAAGAAUAUTT, AS: AUAUUCUUCAGGUAGAGCCTT; PAN3-AS1-siRNA-2# S: GGCCUUCGGUAAAUUAUGATT, AS: UCAUAAUUUACCGAAGGCCTT; BRD4-siRNA-1# S: GCGUUUCCACGGUACCAAATT, AS: UUUGGUACCGUGGAAACGCTT; BRD4-siRNA 2# S: AGCUGAACCUCCCUGAUUATT, AS: UAAUCAGGGAGGUUCAGCUTT; P300-siRNA-1# S: CCGGUGAACUCUCCUAUAATT, AS: UUAUAGGAGAGUUCACCGGTT; P300-siRNA-2# S: GCCUCAAACUACAAUAAAUTT, AS: AUUUAUUGUAGUUUGAGGCTT.

#### 2.3.3 Reverse Transcription and Quantitative Real-Time Polymerase Chain Reaction (RT-qPCR)

SiRNA transfected cells, as described above, or cells treated with the BET bromodomain inhibitors-JQ1-1 μM (S7110, Selleck, Shanghai, China) and I-BET-762-2 μM (S7189, Selleck) for 24 h were utilized to extract total RNA through Trizol (Qiagen, 1023537, Duesseldorf, Germany). After quantifying the RNA, we carried out RNA reverse transcription with a High-Capacity cDNA Kit (Thermo, 4368814, Waltham, MA, USA). The PCR amplification process was tracked in real-time with the UltraSYBR Mixture (CW0957M, CWbiotech, Taizhou, China). α-Tubulin was taken as an internal control for normalization. The primer sequences employed are provided below:

PAN3-AS1-F: 5^′^-TTTTCCTCTTCCTGAGACGGC-3^′^, R: 5^′^-ATCAGGTCTCGTGAGAATTCGG-3^′^; WFDC13-F: 5^′^-CCAAGCAGCGTGTTCTGAAGTA-3^′^, R: 5^′^-ATATGCCTCAGTTGGCAGGC-3^′^; α-Tubulin-F: 5^′^-GAAGCAGCAACCATGCGTGA-3^′^, R: 5^′^-AAGGAATCATCTCCTCCCCCA-3^′^.

#### 2.3.4 Colony Formation and Cell Viability Assays

HCT116 or DLD1 cells were distributed into 6-well plates at a density of 1 × 10^3^ cells/mL after cell transfection. The cells were cultivated for 10 days. Then the colonies underwent fixation in 4% formaldehyde followed by 0.1% crystal violet staining. HCT116 or DLD1 cells were distributed into 96-well plates at a density of 1 × 10^4^ cells/mL after cell transfection. The cellular survival rate was quantified by a Cell Counting Kit-8 (C0038-500, Beyotime, Shanghai, China) following 72-h incubation. The optical density measurement was quantified at 450 nm excitation by means of a microplate reader (Multiskan FC, Thermo Fisher Scientific, Waltham, MA, USA).

#### 2.3.5 Coculture Assays

Human CD8^+^ T cells were obtained from Meisen Chinese Tissue Culture Collections (Hangzhou, China). CD8^+^ T cells were preactivated with anti-CD3 (1:1000) and anti-CD28 antibodies (1:1000) (MULTI SCIENCES, F1100300, and F1102800, Hangzhou, China). HCT116 cells were distributed into 96-well plates at a density of 1 × 10^4^ cells/mL, and CD8^+^ T cells at a density of 1 × 10^5^ cells/mL were cocultured for 48 h. The CD8^+^ T cells were gathered, fixed, and stained with antibodies such as CD3-FITC (1:200) (MULTI SCIENCES, F11003A01), CD8-PE (1:200) (MULTI SCIENCES, F1100802), and PD-1-APC (1:200) (BioLegend, 329907, San Diego, CA, US). Then, CD8^+^ T cells were detected by flow cytometric analysis. The ELISA Kits (MULTI SCIENCES, EK158 and EK1282, Hangzhou, China) were used to quantify Granzyme B and Ki-67 levels in cell culture supernatants.

#### 2.3.6 miRNA Prediction and Analysis

The subcellular position of PAN3-AS1 was forecasted by lncLocator (http://www.csbio.sjtu.edu.cn/bioinf/lncLocator/ (accessed on 01 June 2025)). The miRNAs complementary to the PAN3-AS1 RNA and WFDC13 mRNA 3^′^untranslated region (UTR) were analyzed by the Starbase 3.0 database (https://rnasysu.com/encori/index.php (accessed on 01 June 2025)) and the miRWalk database (http://mirwalk.umm.uni-heidelberg.de/ (accessed on 01 June 2025)). And we investigated miRNA expression profiles using the Starbase 3.0 database (https://rnasysu.com/encori/index.php (accessed on 01 June 2025)).

#### 2.3.7 Assays of Luciferase Activity

The pMIR-REPOPT-PAN3-AS1 (WT or MT) and pMIR-REPOPT-WFDC13-3^′^UTR (Wide type or mutant) plasmids were acquired from OBiO Technology (Shanghai, China). Plasmid DNA was transfected into human 293T cells using X-tremeGENE 9 DNA Transfection Reagent (Roche, 6365779001, Basel, Switzerland). Luciferase activity was recorded by a Dual Luciferase Reporter Kit (Promega, E2920, Madison, WI, USA).

#### 2.3.8 Chromatin Immunoprecipitation (ChIP)

ChIP assays were carried out by the SimpleChIP® Enzymatic Chromatin IP Kit (CST, 9003S, Darmstadt, Germany). 4 × 10^6^ HCT116 or DLD1 cells were fixed with 4% formaldehyde. The genomic DNA was fragmented by 0.5 μL Micrococcal Nuclease. Chromatin immunoprecipitation was performed using H3K27ac antibody (1:500) (Abcam, ab177178, Cambridge, UK) and P300 antibody (1:500) (Abcam, ab275378, Cambridge, UK). Finally, we analyzed the purified DNA using qPCR. The primer sequences for the PAN3-AS1 enhancer region are shown below:

PAN3-AS1-enhancer-F: 5^′^-AGTACTGCCGCTACTACGCT-3^′^; PAN3-AS1-enhancer-R: 5^′^-ACTCCTCCCCGTAGAAGCAA-3^′^.

#### 2.3.9 RNA Immunoprecipitation (RIP)

The RIP assay was executed with a RIPAb+AGO2 kit (EMD Millipore, 03-110, Darmstadt, Germany). 1 × 10^7^ HCT116 or DLD1 cells underwent lysis using the specified buffer and immunoprecipitated with 5 μg of the mIgG or AGO2 antibodies. The total RNA was isolated and further analyzed by RT-qPCR. A miScript PCR system (Qiagen, 218073, Duesseldorf, Germany) was used to determine the miRNA expression level. The isolated RNA was used for RT-qPCR analysis with the same primers described above.

### 2.4 H3H27ac Signal Analysis

We analyzed the H3K27ac signal in the enhancer region of PAN3-AS1 in different COAD cells and the colonic mucosa by using the WashU database (https://epigenomegateway.wustl.edu/browser/ (accessed on 01 June 2025)).

### 2.5 Drug sensitivity Profiling and Molecular Docking

We gained maximum inhibitory concentrations (IC_50_s) of the drug and gene transcriptomics data for 60 malignant cell lines from the CellMiner database (https://discover.nci.nih.gov/cellminer/home.do (accessed on 01 June 2025)). FDA-approved drugs were selected. The correlation coefficient linking WFDC13 abundance and drug IC_50_ values was calculated using R software, and scatter plots were drawn. Subsequently, the spatial structures of the WFDC13 protein and small molecule drugs were obtained from the Protein Data Bank (PDB) (https://www.rcsb.org/ (accessed on 01 June 2025)) and the Pubchem databases (https://pubchem.ncbi.nlm.nih.gov/ (accessed on 01 June 2025)). Docking of proteins and drugs was performed with AutoDockTools software (v4.2.3). We then proceeded to visualize the docking results with PyMOL software (v3.1.6.1). Finally, the two-dimensional structure of protein amino acid residues interacting with small molecule drugs was determined using Ligplus software (v2.3).

### 2.6 Statistical Analysis

Statistical significance was calculated with a two-sided Student’s *t*-test using GraphPad Prism 7 software. The findings are displayed as the means ± standard deviations. If the standard deviations of the results did not agree, a Welch-corrected *t*-test was used. Moreover, statistical tests were executed utilizing R software (4.2.2), using *p* < 0.05 as the significance threshold.

## 3 Results

### 3.1 Expression and Prognostic Analysis across Pan-Cancer

#### 3.1.1 Expression Level Analysis of PAN3-AS1 across Pan-Cancer

**Figure A1:**
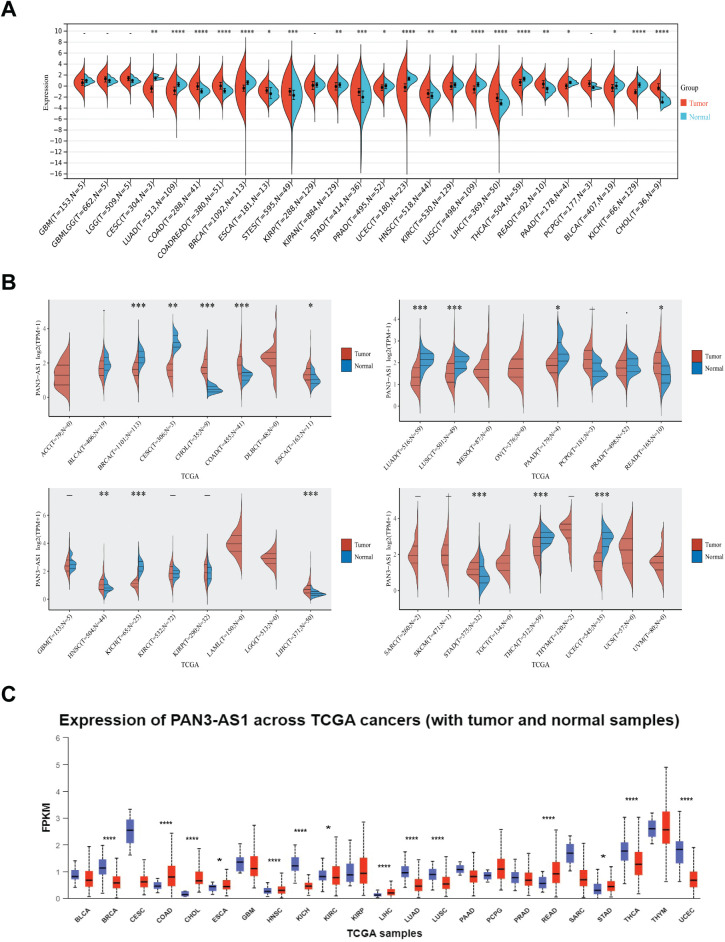
Expression level analysis of PAN3-AS1 in pan-cancer. Expression levels of PAN3-AS1 in normal and tumor tissues were observed from Sangerbox 3.0 (**A**), ACLBI (**B**), and UALCAN (**C**) databases. (*p < 0.05, **p < 0.01, ***p < 0.001, ****p < 0.0001)

**Figure A2:**
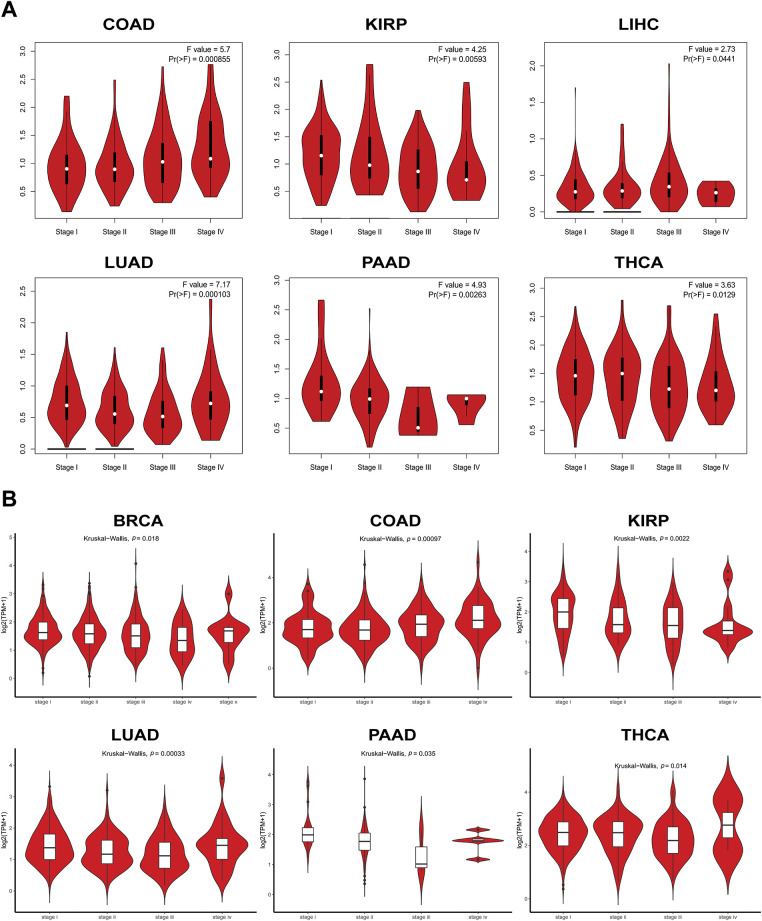
The expression levels of PAN3-AS1 in different pathological stages. (**A,B**) The expression levels of PAN3-AS1 in different pathological stages using GEPIA2 and Lnc2Cancer 3.0 databases

To probe the abundance of PAN3-AS1 in tumors, we investigated the differential expression of PAN3-AS1 across pan-cancer using three databases, namely, Sangerbox 3.0, ACLBI, and UALCAN (Fig. A1A–C). Notably, our combined analysis of the above three databases identified seven cancer types in which PAN3-AS1 expression levels were upregulated, including COAD, cholangiocarcinoma (CHOL), esophageal carcinoma (ESCA), HNSC, liver hepatocellular carcinoma (LIHC), rectum adenocarcinoma (READ), and stomach adenocarcinoma (STAD). However, PAN3-AS1 expression levels were downregulated in six cancer types, namely, breast invasive carcinoma (BRCA), kidney chromophobe (KICH), lung adenocarcinoma (LUAD), lung squamous cell carcinoma (LUSC), thyroid carcinoma (THCA), and uterine Corpus Endometrial Carcinoma (UCEC). In addition, in the GEPIA2 and Lnc2Cancer 3.0 databases, we detected a strong correlation coefficient linking the PAN3-AS1 abundance and clinicopathological stage across a variety of tumor types (Fig. A2A,B). Interestingly, in tumors with high PAN3-AS1 expression, such as COAD and LIHC, PAN3-AS1 abundance was positively linked to clinicopathological stage. On the contrary, PAN3-AS1 abundance was negatively linked to the clinicopathological stage across tumors whose expression was downregulated, such as LUAD and THCA. Thus, PAN3-AS1 has diagnostic value in some malignancies.

#### 3.1.2 Single-Cell Sequencing Data Analysis

**Figure 1:**
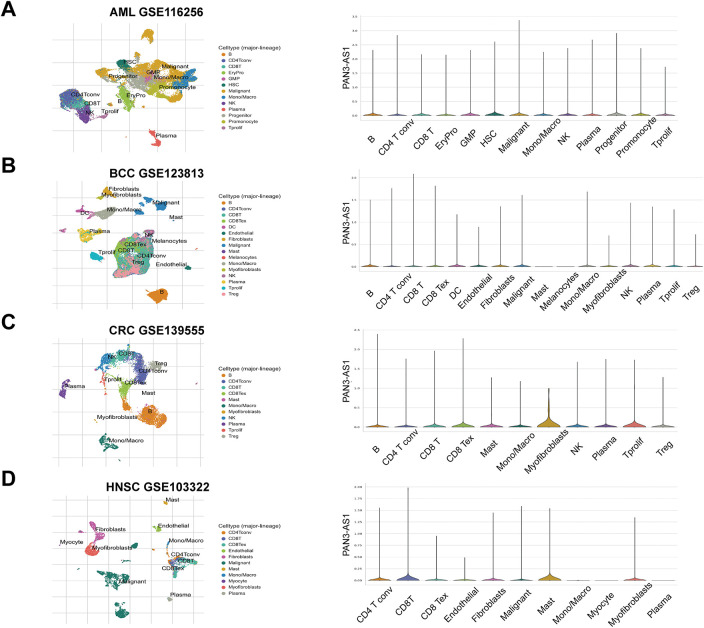

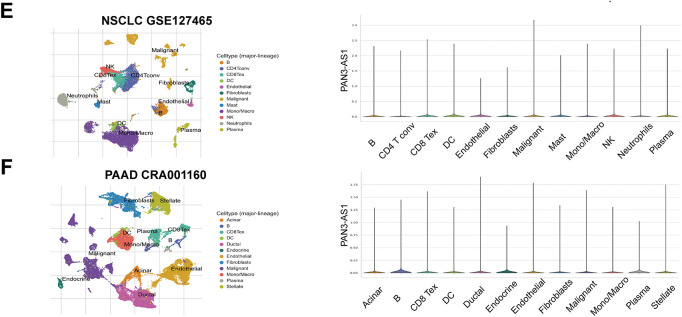
PAN3-AS1 expression levels analysis using the single-cell sequencing data. (**A**) AML-GSE116256, (**B**) BCC-GSE123813, (**C**) CRC-GSE139555, (**D**) HNSC-GSE103322, (**E**) NSCLC-GSE127465, (**F**) PAAD-CRA001160

It is well known that immune clusters emerge as indispensable constituents of the tumor microenvironment (TME) and critically affect the tumor immunotherapy outcome [23]. Recently, single-cell sequencing has been applied to identify gene abundance in immune cells from the tumor microenvironment [24]. Consequently, by means of the TISCH database, we determined the abundance of PAN3-AS1 in a variety of immune cells (Fig. 1). We discovered that in AML, BCC, NSCLC, and PAAD, other than in the malignant cells, PAN3-AS1 is present in many kinds of immune cells. Notably, the highest PAN3-AS1 abundance was detected in the myofibroblasts of the CRC patients. And PAN3-AS1 abundance was highest in the HNSC patients’ mast cells and CD8^+^ T cells. In sum, PAN3-AS1 was enriched in malignant tissues and the tumor microenvironment.

#### 3.1.3 Prognostic Analysis of PAN3-AS1 in Pan-Cancer

**Figure 2:**
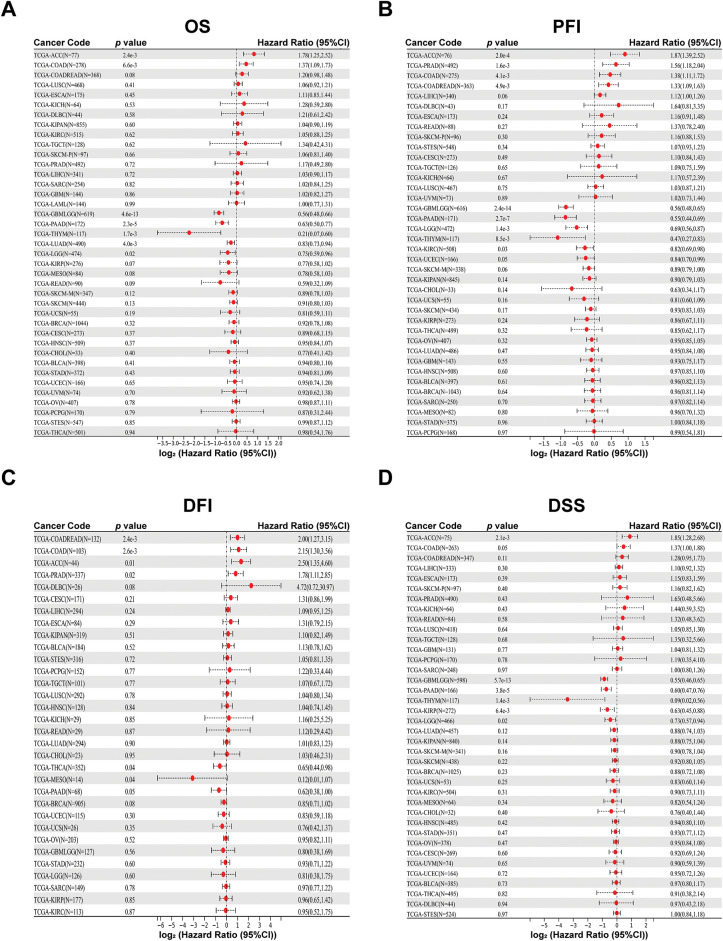
Prognostic analysis for PAN3-AS1 in pan-cancer using Sangerbox 3.0 database. (**A**) OS analysis for PAN3-AS1 in pan-cancer. (**B**) PFI analysis for PAN3-AS1 in pan-cancer. (**C**) DFI analysis for PAN3-AS1 in pan-cancer. (**D**) DSS analysis for PAN3-AS1 in pan-cancer. (OS, overall survival; PFI, progression-free interval; DFI, disease-free interval; DSS, disease-specific survival)

Next, with the Sangerbox 3.0 database, we characterized the relationships between PAN3-AS1 and OS, PFI, DSS, and DFI. The findings supported that the overexpression of PAN3-AS1 was tied to poor OS of patients with two tumor types-adrenocortical carcinoma (ACC) and COAD, in contrast to patients with glioblastoma multiforme (GBM), lower grade glioma (LGG), PAAD, thymoma (THYM) and LUAD, in which patients with elevated PAN3-AS1 levels had favorable OS (Fig. 2A). Furthermore, we found that higher PAN3-AS1 levels was linked to inferior PFI and DFI in three tumor types-ACC, prostate adenocarcinoma (PRAD) and COAD (Fig. 2B,C). However, elevated PAN3-AS1 levels in GBM, LGG, PAAD, THYM, and kidney renal clear cell carcinoma (KIRC) revealed a significant connection to a superior PFI. Higher PAN3-AS1 levels were tied to a better DFI in THCA and mesothelioma (MESO). In addition, elevated PAN3-AS1 levels were linked to inferior DSS in patients with ACC and better DSS in six tumor types patients (Fig. 2D). Overall, in the PAN3-AS1 high-expressing tumor types, such as ACC and COAD, these patients usually have a poor prognosis. If the PAN3-AS1 expression is not significantly different or is downregulated, the patients usually have a good prognosis, such as those with GBMLGG, PAAD, THYM, LUAD, and THCA. Notably, combining the abundance of PAN3-AS1 across pan-cancer, we found that PAN3-AS1 expression was intimately linked to prognostic indicators in COAD. Therefore, the high expression of PAN3-AS1 may be an important indicator of poor prognosis in COAD patients.

### 3.2 Immune Correlation Analysis in Pan-Cancer

#### 3.2.1 Correlation Analysis between PAN3-AS1 Abundance and the TME

**Figure 3:**
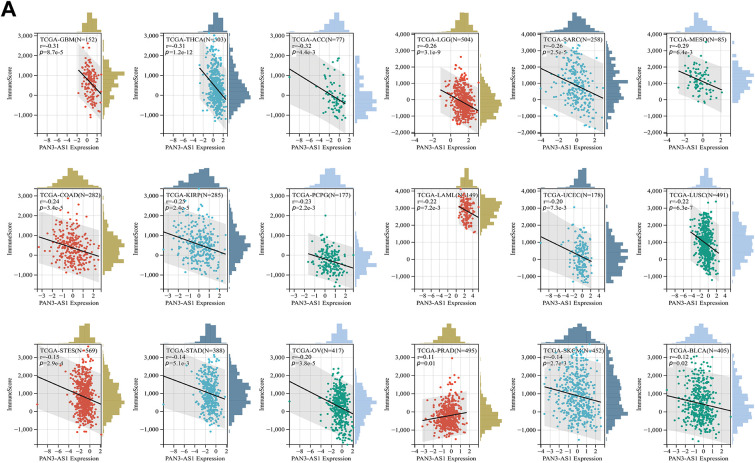

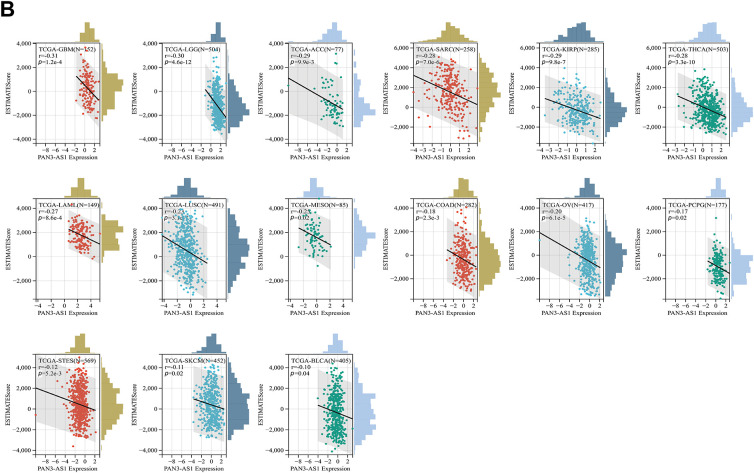
Immune scores analysis for PAN3-AS1 in pan-cancer. Scatter plots of the pertinence between PAN3-AS1 expression and immune score (**A**), ESTIMATE scores (**B**)

**Figure 4:**
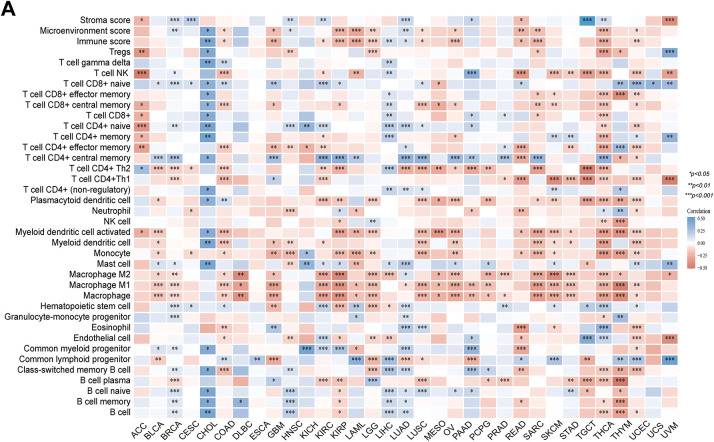

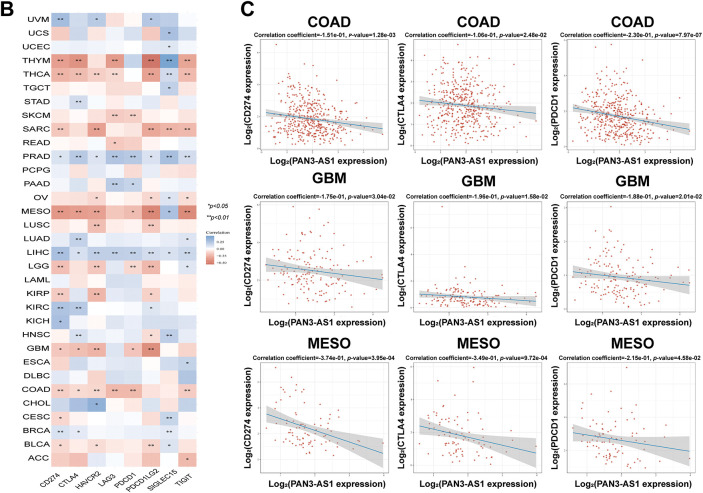
Immune infiltration cells and immune checkpoint genes expression analysis for PAN3-AS1 in pan-cancer. (**A**) The interrelation heatmap between PAN3-AS1 expression and immune-infiltrating cells in pan-cancer (XCELL algorithm). (**B**) The correlation mapping of PAN3-AS1 expression with immune checkpoint gene expression in pan-cancer. (**C**) Scatter plots of the correlation between PAN3-AS1 and CD274 (PD-L1), CTLA4, and PDCD1 (PD-1) expression. (*p < 0.05, **p < 0.01, ***p < 0.001)

Considering that the expression of PAN3-AS1 presents a linkage with the TME, we evaluated the relevance of PAN3-AS1 to immune infiltration scores. We discovered that the PAN3-AS1 expression inversely linked to the immune scores of 18 tumor types and the ESTIMATE scores of 15 tumor types (Fig. 3). In addition, by using the XCELL algorithm, we plotted a correlation heatmap between the abundance of PAN3-AS1 and immune infiltrating cells in pan-cancer (Fig. 4A). The findings revealed that PAN3-AS1 expression inversely linked to immune cells infiltration in the majority of tumor types. Studies have shown that tumors can engage in immune escape via immune checkpoints [25]. By using the ACLBI database, we analyzed the relevance of PAN3-AS1 to immune checkpoint genes. Ultimately, we identified PAN3-AS1-immune checkpoints co-expression modules in PRAD and LIHC, but opposite correlations in COAD, GBM, MESO, THCA, and THYM (Fig. 4B). In Fig. 4C, PAN3-AS1 expression was inversely linked to CD274 (PD-L1), PDCD1 (PD-1), and CTLA4 expression simultaneously in COAD, GBM, and MESO. Collectively, this evidence implies that AN3-AS1 expression may be associated with a “cold” immune microenvironment in many tumor types.

#### 3.2.2 PAN3-AS1 Is a Biomarker to Predict Immunotherapy Efficacy

**Figure 5:**
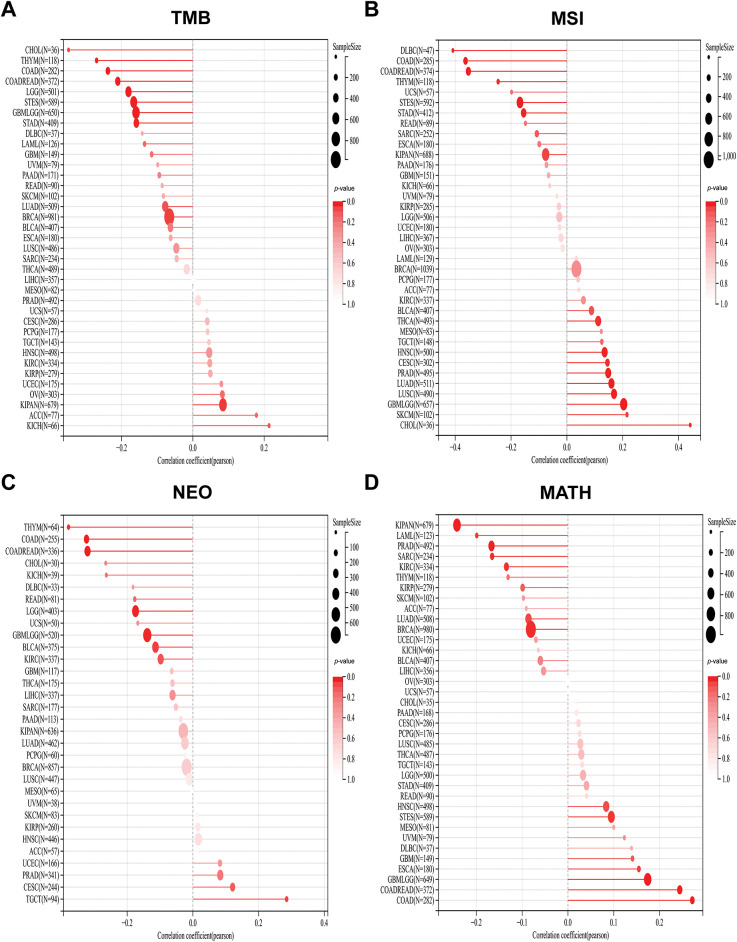
Stick charts of the correlation between PAN3-AS1 expression and TMB, MSI, NEO, and MATH in pan-cancer. (**A**) The TMB correlation with PAN3-AS1 in pan-cancer. (**B**) The MSI correlation with PAN3-AS1 in pan-cancer. (**C**) The NEO correlation with PAN3-AS1 in pan-cancer. (**D**) The MATH correlation with PAN3-AS1 in pan-cancer. (TMB, Tumor mutational burden, MSI, Microsatellite instability, NEO, Neoantigens, MATH, Mutant-allele tumor heterogeneity)

**Figure 6:**
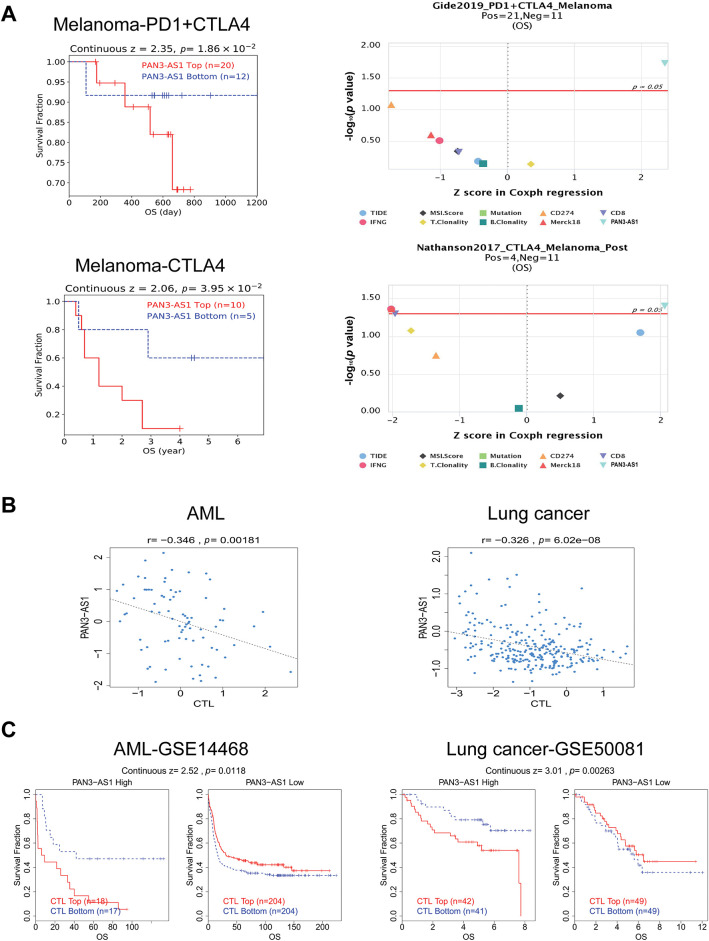
PAN3-AS1 predicted immunotherapy efficacy. (**A**) The OS analysis of PAN3-AS1 high or low expressed melanoma patients receiving PD1 and CTLA4 antibodies. (**B**) The relevance between PAN3-AS1 expression and CTL levels in AML and lung cancer. (**C**) The OS analysis of AML and lung cancer patients with different PAN3-AS1 expression and CTL levels. (OS, overall survival; CTL, cytotoxic T cell; AML, acute myeloid leukemia)

Studies have shown that indicators of genomic heterogeneity could be used as potential biomarkers for immunotherapy efficacy [26–28]. Well, does PAN3-AS1 have an expression correlation with these biomarkers? We characterized the relevance of PAN3-AS1 to TMB, MSI, NEO, and MATH. The results demonstrated that PAN3-AS1 expression inversely linked to the TMB in nine tumor types-CHOL, THYM, COAD, CRC, LGG, stomach and esophageal carcinoma (STES), GBM, STAD and BRCA (Fig. 5). We observed an inverse correlation linking PAN3-AS1 expression to the MSI in six neoplasms, namely diffuse large B cell lymphoma (DLBC), COAD, CRC, THYM, STES, and STAD, but a positive relationship in ten tumor types, such as THCA, HNSC, cervical squamous cell carcinoma and endocervical adenocarcinoma (CESC), PRAD, LUAD, LUSC, GBM, LGG, SKCM, and CHOL. Moreover, PAN3-AS1 levels are inversely linked to NEO in THYM, COAD, CRC, LGG, GBM, and bladder urothelial carcinoma (BLCA). In terms of tumor heterogeneity, we observed an inverse correlation linking PAN3-AS1 expression to the MATH in the pan-kidney cohort (KICH+KIRC+KIRP) (KIPAN), acute myeloid leukemia (LAML), PRAD, sarcoma (SARC), KIRC, and BRCA, but a positive correlation in many tumor types, such as STES, ESCA, GBM, LGG, CRC, and COAD. Evidence suggests that the immune heterogeneity of the TME also interferes with immunotherapeutic outcomes [29]. Therefore, we supposed that PAN3-AS1 might be a reliable biomarker of immunotherapy efficacy in many tumor types. Subsequently, overall survival (OS) analysis indeed confirmed that cases exhibiting high PAN3-AS1 levels had poor survival rates when they received PD1 and CTLA4 antibody treatment in melanoma (Fig. 6A). PAN3-AS1 showed a better predictive value than the other biomarkers (*p* < 0.05). This implies that melanoma cases exhibiting high PAN3-AS1 levels were not suitable for immunotherapy. In addition, we found that PAN3-AS1 abundance inversely linked to the number of cytotoxic T cells (CTL) in AML and lung cancer (Fig. 6B). More importantly, the K-M curves demonstrated a negative correlation between PAN3-AS1 expression and CTL function (Fig. 6C), implying that high PAN3-AS1expression may impair CTL function in these tumors. The accumulated evidence supports PAN3-AS1 as a promising candidate for both predictive biomarker and immunotherapy target.

### 3.3 Downstream Regulatory Mechanism for PAN3-AS1 in COAD

#### 3.3.1 Functional Enrichment Analysis of PAN3-AS1 in COAD

**Figure 7:**
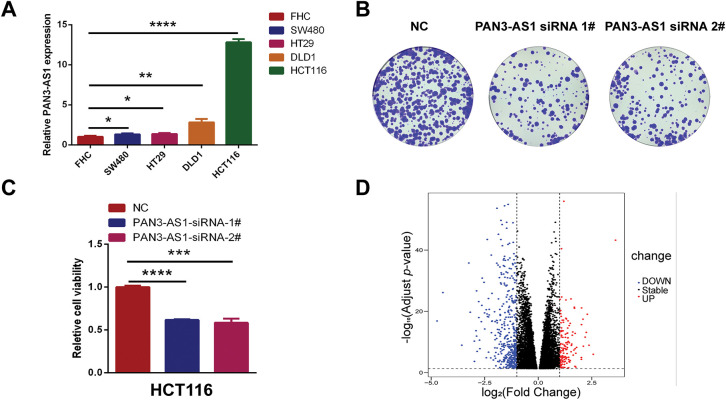

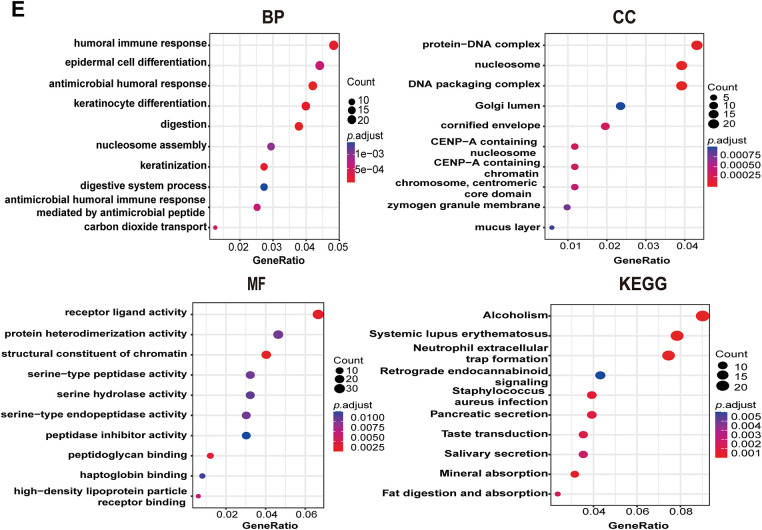
Functional enrichment analysis of PAN3-AS1 in COAD. (**A**) The relative expression levels of PAN3-AS1 in colon cancer cells compared with normal colon epithelial cell-FHC. (**B**) The colony formation ability of HCT116 cells was measured after PAN3-AS1 knockdown by siRNA. (**C**) Relative cell viability of HCT116 cells was measured after PAN3-AS1 knockdown by siRNA. (**D**) The volcano of PAN3-AS1-related differentially expressed genes (KRDEGs) in COAD. (**E**) GO and KEGG analysis of KRDEGs in COAD. (COAD, colon cancer, GO, gene ontology, BP, biological process, CC, cellular components, MF, molecular function, KEGG, Kyoto encyclopedia of genes and genomes) (*p < 0.05, **p < 0.01, ***p < 0.001, ****p < 0.0001)

**Figure A3:**
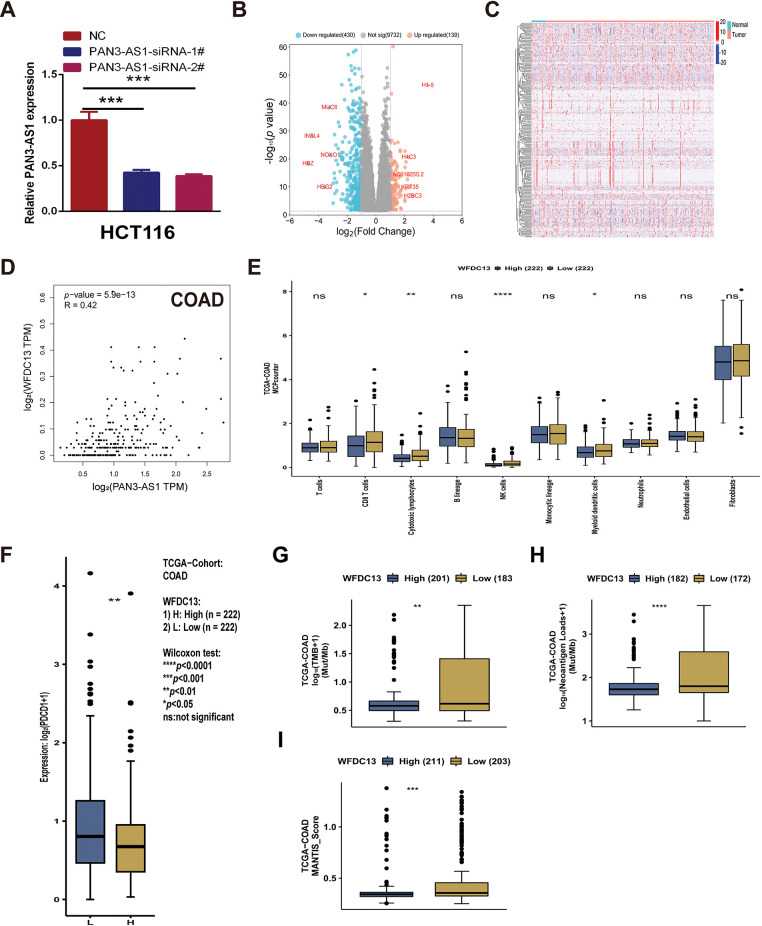
PAN3-AS1 regulated the immune microenvironment by mediating WFDC13 expression in COAD. (**A**) The interference efficacy of PAN3-AS1 siRNA was measured by RT-qPCR. (**B**) The volcano of PAN3-AS1-related differentially expressed genes (KRDEGs) in COAD. (**C**) The heatmaps of 287 KRDEGs in the TCGA-COAD datasets. (**D**) The correlation between the expression of PAN3-AS1 and WFDC13 in COAD. (**E**) The correlation between WFDC13 expression and immune cell infiltration in COAD using the CAMOIP database. (**F**–**I**) The differences in PDCD1 expression levels, TMB, neoantigen, and MANTIS score between WFDC13 high and low expression groups in COAD using the CAMOIP database. (COAD, colon cancer, TMB, Tumor mutational burden, *p < 0.05, **p < 0.01, ***p < 0.001, ****p < 0.0001, ns, no significance)

The above data revealed that PAN3-AS1exhibited consistent elevation in seven malignancies: COAD, CHOL, ESCA, HNSC, LIHC, READ, and STAD. Remarkably, among them, PAN3-AS1 expression was intimately tied to the four prognostic indicators only in COAD. Therefore, based on the key role of PAN3-AS1 in the expression, stage, prognosis, and tumor immunological microenvironment of COAD, we were interested in probing the biological function of PAN3-AS1 in COAD. First, we detected PAN3-AS1 abundance in COAD cell lines. We observed that PAN3-AS1 expression was highest in HCT116 cells than in the normal colon epithelial cell-FHC (*p* < 0.0001) (Fig. 7A). By using two independent siRNAs targeting PAN3-AS1 (Fig. A3A), the colony formation ability and cell viability of HCT116 cells were obviously impaired after knockdown of PAN3-AS1 (Fig. 7B,C). These results demonstrated that PAN3-AS1 was indispensable for cellular multiplication in COAD. Next, we analyzed the DEGs depending on the median abundance of PAN3-AS1 in the TCGA-COAD cohort (Figs. 7D and A3B). We obtained 569 differentially expressed genes. Moreover, among them, 287 genes were also differentially expressed in the TCGA-COAD dataset. Therefore, we mapped the heatmap of these genes (Fig. A3C). Subsequently, the above 569 DEGs were characterized by GO and KEGG analysis (Fig. 7E). The biological process (BP) results suggest that PAN3-AS1 is predominantly engaged in humoral immune response. KEGG enrichment analysis showed predominantly alcoholism and neutrophil extracellular trap formation, etc. In summary, we inferred that the role of PAN3-AS1 in COAD was related to the immune response process.

#### 3.3.2 PAN3-AS1 Regulated the Immune Response by Mediating WFDC13 Expression in COAD

**Figure 8:**
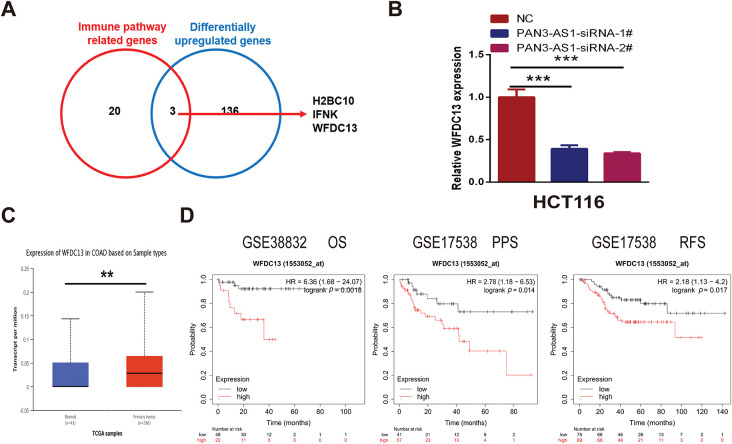

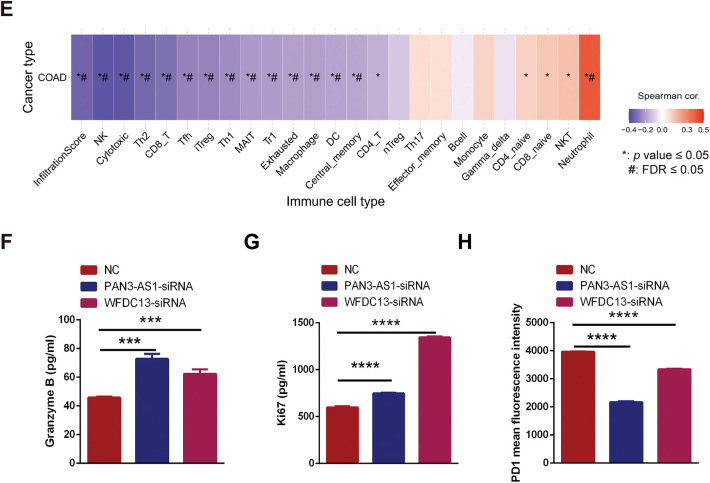
PAN3-AS1 regulated the immune response by mediating WFDC13 expression in COAD. (**A**) The Venn diagrams of the intersection of immune pathway-related genes and differentially upregulated genes. (**B**) The expression of WFDC13 mRNA after PAN3-AS1 silencing was determined using the RT-qPCR assay. (**C**) The WFDC13 expression levels in the normal and COAD samples using TCGA data. (**D**) Prognostic analysis for WFDC13 in COAD using Kaplan Meier-plotter database. (**E**) The correlation between the GSVA score of WFDC13 and immune cell infiltration in COAD using the GSCA database. (**F**,**G**) Granzyme B and Ki67 expression in the co-culture supernatant was measured using ELISA. (**H**) The PD1 means fluorescence intensity of CD8^+^ T cells was measured using flow cytometry. (*p < 0.05, **p < 0.01, ***p < 0.001, ****p < 0.0001, #, FDR < 0.05)

**Figure A4:**
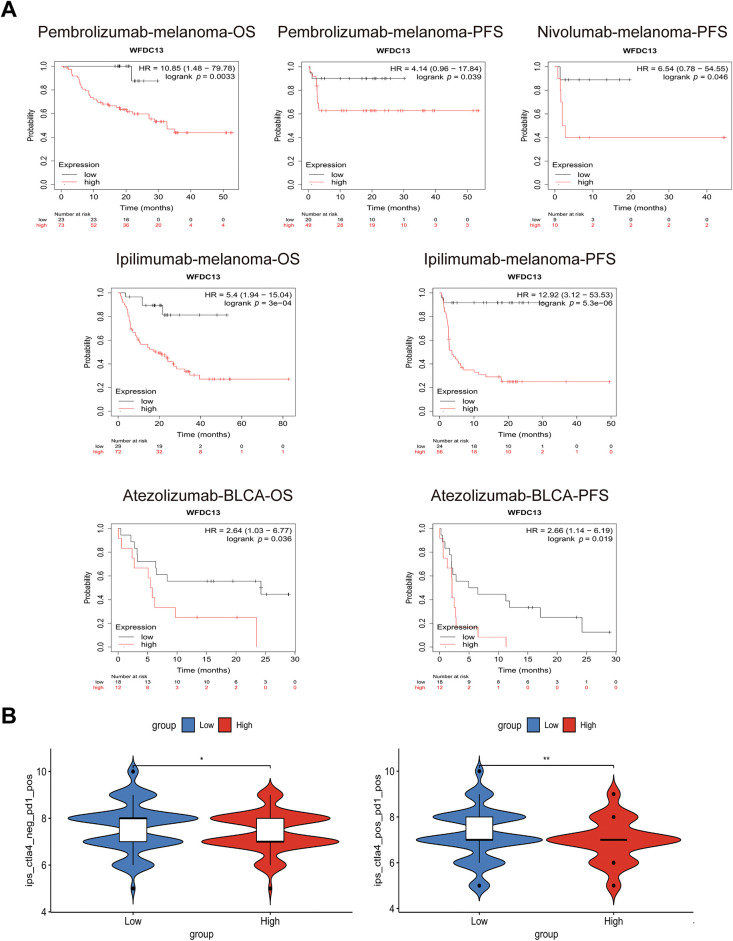
WFDC13 is a biomarker for predicting the immunotherapy efficiency. (**A**) The OS and PFS analysis of WFDC13 high or low expressed cancer patients receiving PD1, PDL1, and CTLA4 antibodies. (**B**) Comparison of the IPS between high and low WFDC13 expression groups in COAD. (IPS, Immunophenoscore, *p < 0.05, **p < 0.01)

**Figure A5:**
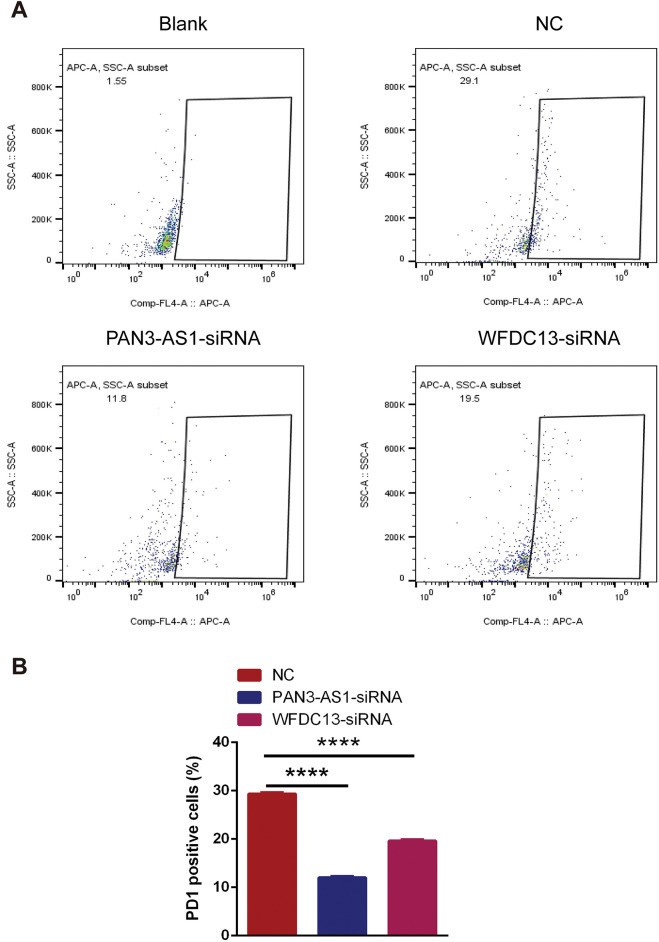
The PD1-positive rates of CD8^+^ T cells were measured using flow cytometry. (**A**) Representative flow cytometry analysis results. (**B**) Statistical chart (****p < 0.0001)

We wondered about the regulatory mechanism of PAN3-AS1 on the immune response in COAD. In Fig. 7D, we got 139 differentially upregulated genes in COAD. In Fig. 7E, 23 genes were involved in the humoral immune response of the biological process. Therefore, we conducted the intersection of these two gene sets and obtained three genes-H2BC10, IFNK, and WFDC13, which not only belonged to the differentially upregulated genes but also the humoral immune response-related genes (Fig. 8A). However, only WFDC13 abundance positively linked to PAN3-AS1 abundance in the TCGA-COAD dataset (Fig. A3D). More importantly, our assay verified that WFDC13 mRNA levels significantly decreased after PAN3-AS1 silencing (Fig. 8B), indicating that PAN3-AS1 could indeed regulate WFDC13 expression in COAD. Furthermore, the WFDC13 expression levels were increased and correlated with poor prognosis in the COAD datasets (Fig. 8C,D). What about the roles of WFDC13 in the COAD immune microenvironment? Is it the same as that of PAN3-AS1? Interestingly, GSCA platform analysis indicated an inverse correlation between WFDC13 abundance and most of the immune cell abundance in COAD (Fig. 8E). And investigation of the CAMOIP database also highlighted that WFDC13 abundance inversely linked to the immune cell infiltration, like CD8^+^ T cells and NK cells (Fig. A3E). In accordance with the above PAN3-AS1 results, WFDC13 expression is also inversely linked to PD1 abundance level, TMB, neoantigen, and MANTIS scores in COAD (Fig. A3F–I). Meanwhile, the Kaplan-Meier Plotter database showed that WFDC13, highly expressed in melanoma and BLCA patients, indeed had a poor prognosis when they received anti-PD1, anti-PDL1, or anti-CTLA4 antibodies (Fig. A4A). Because the immunotherapy data for COAD are absent in this database, we calculated the Immunophenoscore (IPS), which is another method employed to evaluate the tumor immunogenicity now [30]. The WFDC13 high-expression group exhibited diminished IPS levels, suggesting that WFDC13 high-expressed COAD patients may have a poor prognosis when receiving PD1 and CTLA4 antibodies (Fig. A4B). Could Whether PAN3-AS1/WFDC13 axis directly regulate the immune cell activity? By a coculture system, we found that CD8^+^ T cells with PAN3-AS1 or WFDC13 knockdown tumor cells exhibited higher granzyme B production and enhanced Ki67 activity (Fig. 8F,G). Notably, these CD8^+^ T cells also exhibited downregulated PD1 mean fluorescence intensity and positive rates (Figs. 8H and A5). These data suggest that the PAN3-AS1/WFDC13 axis indeed could directly inhibit CD8^+^ T cell activity. Thus, PAN3-AS1 may regulate the immune response by mediating WFDC13 expression in COAD.

#### 3.3.3 PAN3-AS1 Promoted WFDC13 Expression by Competitively Binding to hsa-miR-423-5p

**Figure 9:**
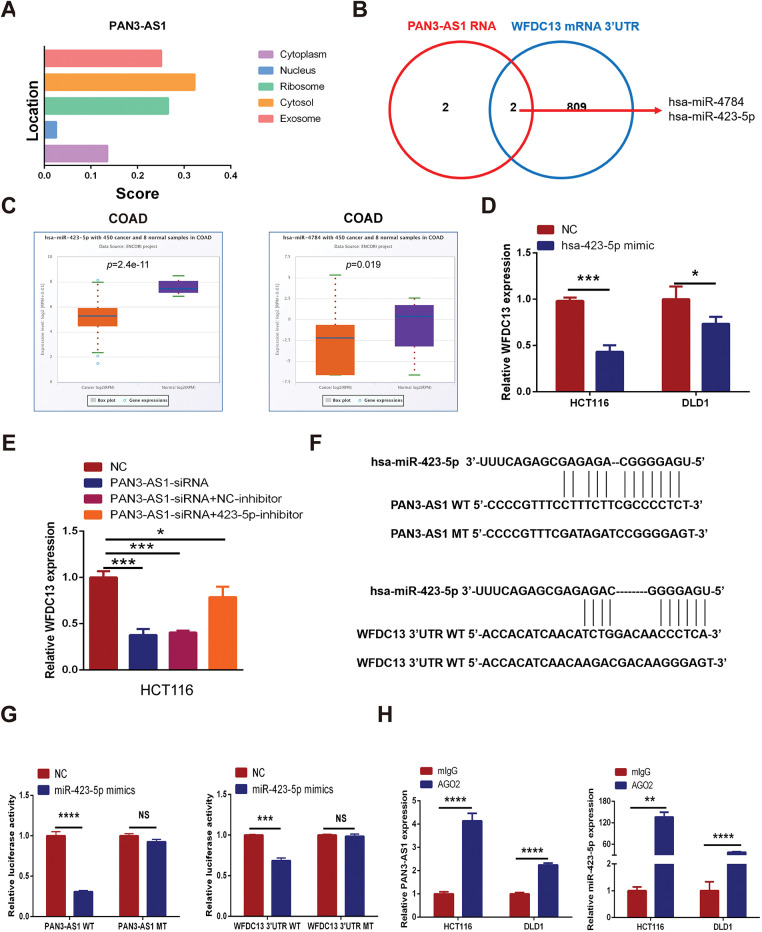
PAN3-AS1 promoted WFDC13 expression by competitively binding to miR-423-5p in COAD. (**A**) The subcellular localization of PAN3-AS1 was predicted by the lncLocator database. (**B**) The Venn diagrams of a series of miRNAs complementary to PAN3-AS1 RNA and the WFDC13 3^′^UTR predicted by bioinformatic tools. (**C**) The relative expression of miR-423-5p and miR-4784 in COAD using the Starbase 3.0 database (https://rnasysu.com/encori/index.php (accessed on 01 June 2025)). (**D**) The effect of miR-423-5p mimics on WFDC13 mRNA expression levels. (**E**) The effect of miR-423-5p inhibitors on PAN3-AS1 knockdown-induced reduction of WFDC13 expression. (**F**) Schematic representation of the predicted target site for miR-423-5p in PAN3-AS1 RNA and the WFDC13 3^′^UTR. (**G**) Dual luciferase assays of PAN3-AS1 RNA or WFDC13 3^′^UTR constructions with intact or mutated seed sequences for miR-423-5p. (**H**) The relative expression levels of PAN3-AS1 and miR-423-5p by RNA immunoprecipitation assays. (*p < 0.05, **p < 0.01, ***p < 0.001, ****p < 0.0001, NS, no significance)

How does PAN3-AS1 regulate the expression of WFDC13 in COAD? The subcellular localization of lncRNAs is generally closely related to their function. By using lncLocator, we estimated that PAN3-AS1 was predominantly localized in the cytosol (Fig. 9A). LncRNAs in the cytosol regulate mRNA stability or the translational process through the ceRNA mechanism [31]. Therefore, by using two databases, Starbase 3.0 and miRWalk, we screened out two miRNAs complementary to the PAN3-AS1 RNA and WFDC13 mRNA 3^′^UTR concurrently (Fig. 9B). In the Starbase 3.0 database (https://rnasysu.com/encori/index.php (accessed on 01 June 2025)), the abundance of miR-423-5p and miR-4784 was diminished in COAD (Fig. 9C). Interestingly, forced miR-423-5p abundance dramatically restrained the formation of WFDC13 (Fig. 9D). Moreover, the PAN3-AS1 knockdown-induced reduction in WFDC13 expression was partially extricated by the miR-423-5p inhibitors (Fig. 9E). In addition, we mapped the candidate binding sites for miR-423-5p in the PAN3-AS1 RNA and WFDC13 mRNA 3^′^UTR (Fig. 9F). Dual luciferase assays validated miR-423-5p binding to PAN3-AS1 RNA and WFDC13 mRNA 3^′^UTR and inhibited their activities. Nevertheless, once the target sites in the PAN3-AS1 RNA and WFDC13 mRNA 3^′^UTR were mutated, then miR-423-5p could not inhibit their activities (Fig. 9G). RIP assays were carried out with anti-Ago2 antibodies. Anti-Ago2 immunoprecipitation enriched more PAN3-AS1 and miR-423-5p (Fig. 9H). In sum, PAN3-AS1 could promote WFDC13 formation in COAD by competitively binding to miR-423-5p.

### 3.4 Upstream Regulatory Mechanism for PAN3-AS1 Overexpression in COAD

#### 3.4.1 P300-Mediated Enhancer Activity Modulated PAN3-AS1 Expression

**Figure 10:**
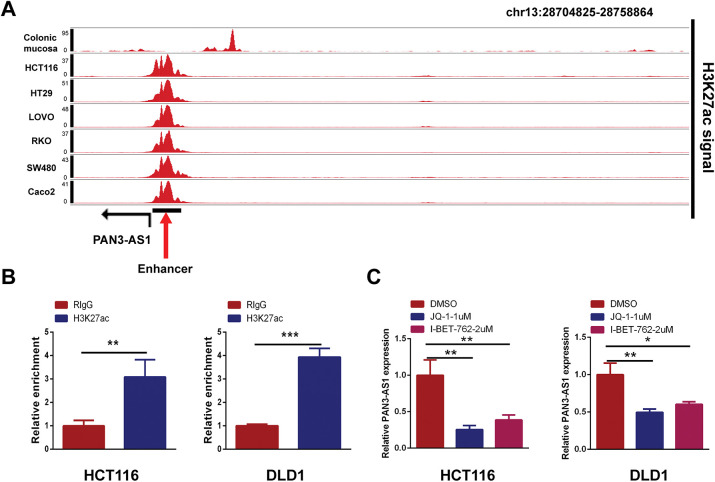

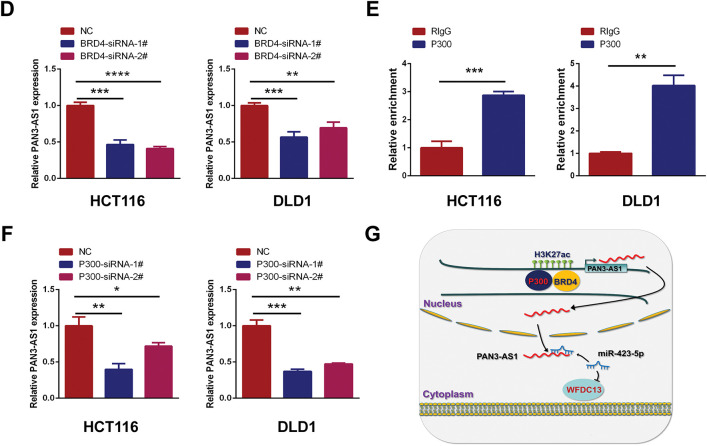
P300-mediated enhancer activity modulated PAN3-AS1 expression. (**A**) Gene tracks of H3K27ac ChIP-seq occupancy at PAN3-AS1 gene loci in different COAD cell lines. (**B**) The relative enrichment levels of H3K27ac to the PAN3-AS1 enhancer region by ChIP-qPCR. (**C**) The relative PAN3-AS1 expression after the treatment with BRD4 inhibitors JQ1 (1 μM) or I-BET-762 (2 μM), by qPCR. (**D**) The relative PAN3-AS1 expression after BRD4 knockdown by RT-qPCR. (**E**) The relative enrichment levels of P300 to the PAN3-AS1 enhancer region by ChIP-qPCR. (**F**) The relative PAN3-AS1 expression after P300 knockdown by RT-qPCR. (**G**) Working model. (*p < 0.05, **p < 0.01, ***p < 0.001, ****p < 0.0001)

**Figure A6:**
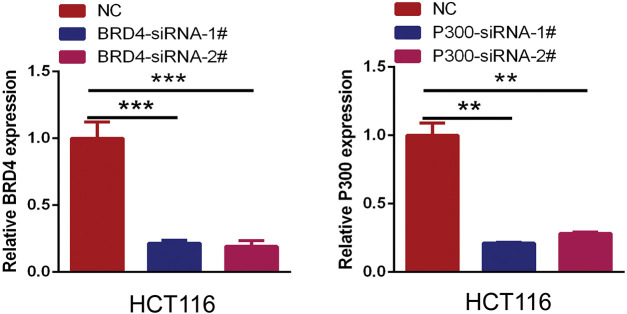
The interference efficacy of BRD4 and P300 siRNA was measured by RT-qPCR. (**p < 0.01, ***p < 0.001)

H3K27ac signal is the most commonly used biomarker for enhancer activity. When using the WashU database, we unexpectedly observed a robust H3K27ac signal in the enhancer region of PAN3-AS1 in different COAD cells, but not in the colonic mucosa (Fig. 10A). By ChIP-qPCR, we confirmed that H3K27ac was significantly enriched in the enhancer region of PAN3-AS1 (Fig. 10B). BRD4 could recognize H3K27ac signals in the enhancer region to regulate gene transcription [32]. Subsequently, exposure to BRD4 inhibitors or siRNAs may disrupt the formation of enhancer [33], leading to lower expression levels of PAN3-AS1 in COAD cells (Figs. 10C,D and A6). It has been discovered that the enhancer activity is dynamically activated by acetyltransferase P300 [34]. And our ChIP-qPCR assay confirmed that P300 was also significantly enriched in the enhancer region of PAN3-AS1 (Fig. 10E). Knocking down P300 reduced the expression of PAN3-AS1 in COAD cells (Fig. 10F). In conclusion, the above results indicated that the enhancer activity modulated the expression of PAN3-AS1 in COAD (Fig. 10G). Ultimately, we experimentally verified the impact of enhancer activity on PAN3-AS1 expression.

#### 3.4.2 WFDC13 Protein-Sensitive Drug Prediction

**Figure 11:**
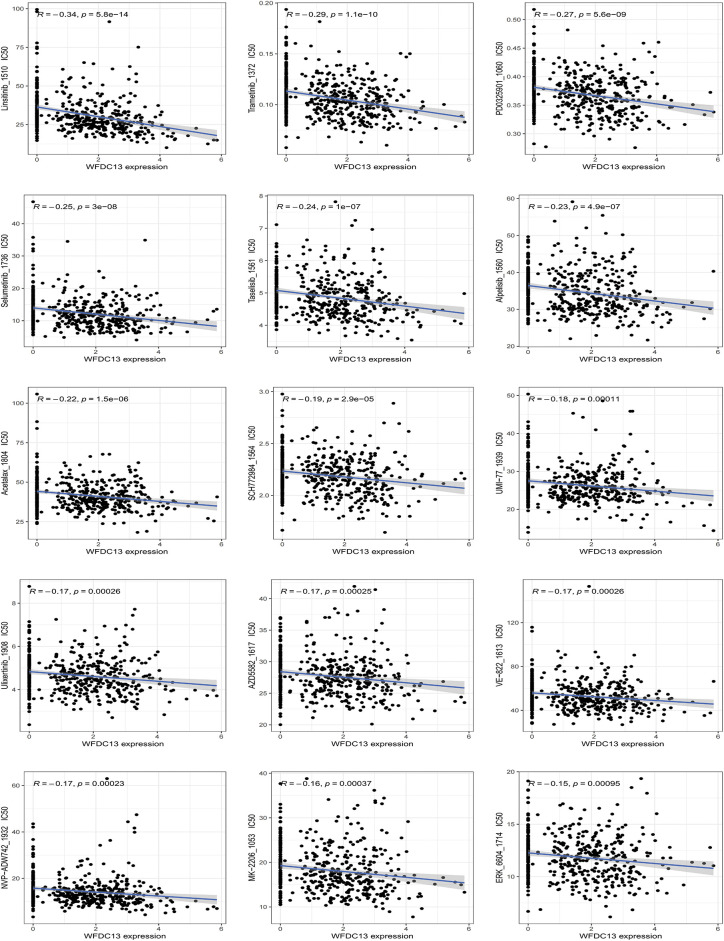
The relevance between WFDC13 expression and drug response using the CellMiner database

**Figure 12:**
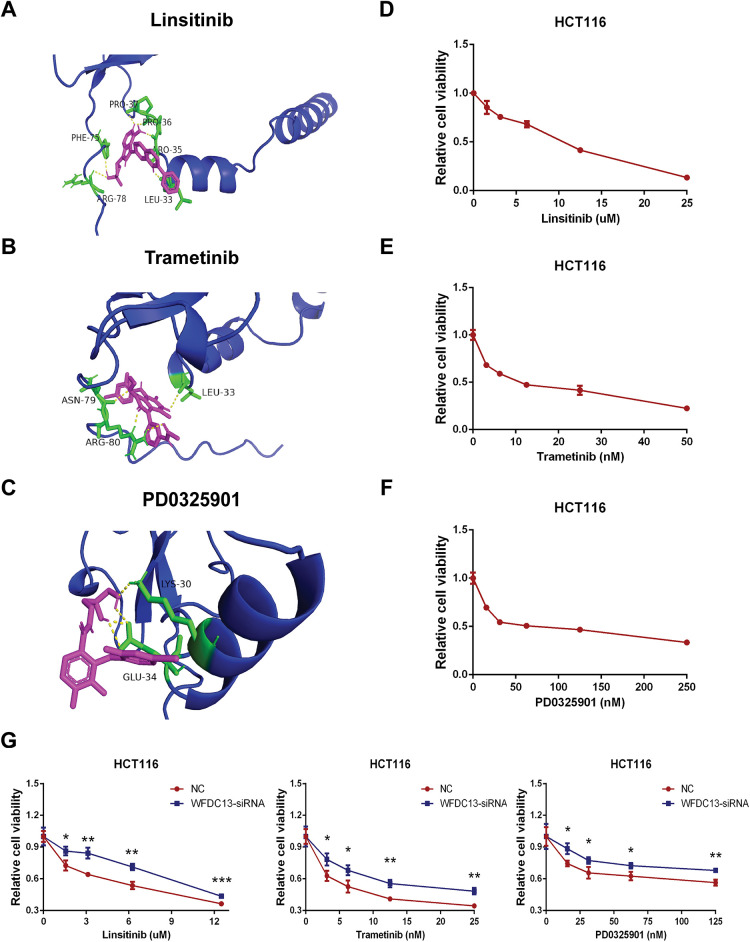
Screening sensitive drugs for the WFDC13 protein. (**A–C**) The optimal docking space conformation of the drugs to the WFDC13 protein. (**D**–**F**) The cell viability was measured by the CCK8 assay. (**G**) The cell viability was measured by the CCK8 assay after knocking down WFDC13 by siRNA. (*p < 0.05, **p < 0.01, ***p < 0.001)

**Figure A7:**
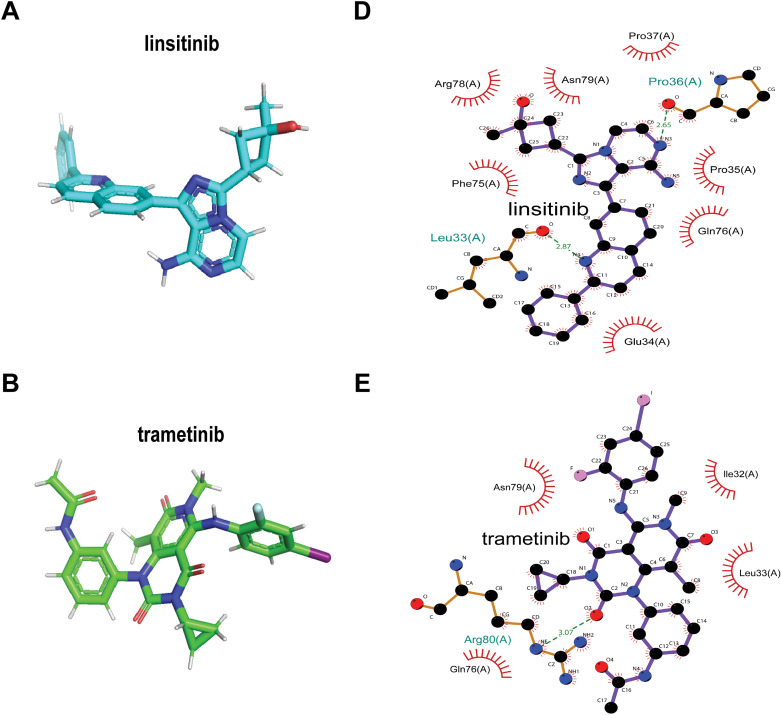

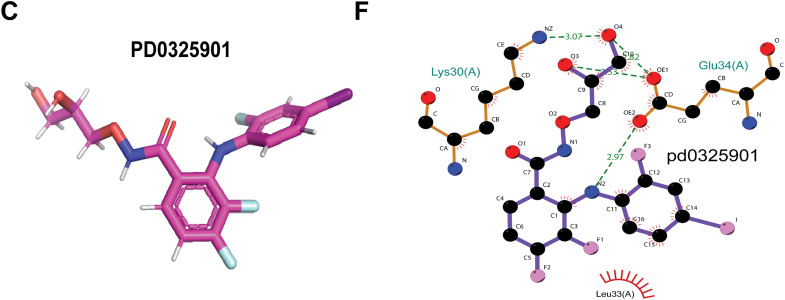
Screening sensitive drugs for the WFDC13 protein. (**A–C**) The structures of predicted sensitive drugs for WFDC13. (**D**–**F**) The interaction force between the WFDC13 protein and the drugs

Finally, our goal was to screen sensitive drugs targeting WFDC13. FDA-approved drugs were downloaded to analyze drugs sensitive to the WFDC13 protein through the CellMiner database. Fig. 11 shows the scatter plots of drugs with correlation to the WFDC13 expression. Afterwards, we downloaded the WFDC13 protein structure from the PDB database. Then we selected three drugs with the highest correlation coefficients, namely, linsitinib, trametinib, and PD0325901. We gained their molecular structures from PubChem (Fig. A7A–C). Next, using AutoDock and PyMOL software, we performed molecular docking and visualized the interactions between the WFDC13 protein and three small-molecule drugs, respectively (Fig. 12A–C and Table A1). Moreover, the two-dimensional structure of the WFDC13 protein interacting with three drugs was determined by Ligplus software (Fig. A7D–F). Finally, we tested the effects of these three drugs by *in vitro* assay. We found that these three drugs could significantly inhibit the cell viability of HCT116 cells (Fig. 12D–F). Is this effect functionally dependent on WFDC13? To validate this, we knocked down WFDC13 by siRNA and discovered that the cell viability was partially rescued, suggesting that WFDC13 may be a target of these three drugs (Fig. 12G).

## 4 Discussion

### 4.1 PAN3-AS1 as a Diagnostic/Prognostic Marker

A few studies have reported that PAN3-AS1 has an essential effect on the progression of pancreatic cancer [14,15]. Ping et al. found that PAN3-AS1 was one of the ferroptosis-related lncRNAs that were responsible for patients’ prognosis and that PAN3-AS1 could be a possible therapeutic target for pancreatic cancer. However, current studies are limited to the effects of PAN3-AS1 only in pancreatic cancer and lack an overall analysis of its roles in pan-cancer. By performing a comprehensive pan-cancer analysis of PAN3-AS1, this study revealed its similarities and differences across different tumors. Our findings showed that PAN3-AS1 was highly expressed in seven tumors. Meanwhile, PAN3-AS1 expression was correlated with clinicopathological stages, suggesting that PAN3-AS1 may be a unique diagnostic biomarker in tumors. Overall, in the PAN3-AS1 highly expressed tumor types, such as ACC and COAD, these patients usually have a poor prognosis. If the PAN3-AS1 expression has no difference or is downregulated, the patients usually have a good prognosis, such as those with GBMLGG, PAAD, THYM, LUAD, and THCA. Therefore, PAN3-AS1 may be an oncogene. Notably, our pan-cancer analysis revealed that PAN3-AS1 was tied to a better prognosis in pancreatic cancer patients. This difference may result from Ping et al.’s signature. PAN3-AS1 itself is an indicator of good prognosis because it is downregulated in PAAD. But the signature containing PAN3-AS1 is an indicator of poor prognosis. This may be caused by the signature, which usually weights the expression levels of several genes. Unexpectedly, our study found that all the prognostic predictors were poor in COAD patients with high expression of PAN3-AS1, suggesting that PAN3-AS1 might be a reliable biomarker for COAD patients’ prognosis analysis.

### 4.2 Roles of PAN3-AS1 in the TME

The development of immunotherapy has led to great progress in tumor treatment. Advantageous results have been obtained in the clinical application of advanced cell therapy and immune checkpoint inhibitors (ICIs) [35]. Studies have already shown favorable clinical outcomes with immune checkpoint inhibitors in melanoma patients. However, a proportion of patients still do not benefit in the long term, suggesting the need to find more appropriate biomarkers and more suitable targets for immunotherapy [36]. Immune infiltrating cells in the TME are the cellular basis for immunotherapy. Thus, deeper insight into immune-infiltrating cells (IICs) within the TME could be crucial for predicting immunotherapy efficacy. The advancement of single-cell sequencing technology offers us a robust tool to decipher the TME [37]. According to the single-cell sequencing data, our study found that PAN3-AS1 expression was identified to be enhanced in immune cells from multiple cancer types. Subsequently, PAN3-AS1 abundance was proven to be inversely tied to immune scores and immune cell infiltration in a variety of tumor categories, suggesting that a possible suppressive immune microenvironment is associated with high PAN3-AS1 expression. In addition, we detected that PAN3-AS1 expression showed a negative correlation with that of CD274 (PD-L1), PDCD1 (PD1), and CTLA4 in many tumors. These findings demonstrated that patients who highly expressed PAN3-AS1 might not be appropriate for treatment with ICIs. Existing studies suggest that MSI and TMB can anticipate immunotherapy outcomes in specific tumor types [38,39]. Interestingly, we found that PAN3-AS1 abundance is inversely linked to TMB and MSI in many tumor categories, which also means that these patients respond poorly to immunotherapy. More importantly, our study revealed that patients with elevated PAN3-AS1 levels were linked to inferior outcomes when PD1 and CTLA4 inhibitors were used to treat melanoma patients. All of the above results support PAN3-AS1 as a promising candidate for an immunotherapy predictive biomarker. By this prediction, it facilitates precise patient selection for immunotherapy to get rid of unnecessary expression and side effects. Given that PAN3-AS1 expression was associated with an inhibitory immune microenvironment, we further discovered that PAN3-AS1 may impair cytotoxic T cell-mediated antitumor immunity, thereby promoting immune escape. This data support PAN3-AS1 as a promising candidate for an immunotherapy target. Whether targeting PAN3-AS1 could improve the immunotherapy efficacy? By a coculture assay, our study validated that PAN3-AS1 could directly inhibit CD8^+^ T cell activity. With respect to the effect on immunotherapy efficacy, additional *in vitro* and *in vivo* approaches are required to substantiate our assumption.

### 4.3 Molecular Mechanism Exploration in COAD

PAN3-AS1 is a valuable biomarker for COAD’s diagnosis and prognosis. Meanwhile, PAN3-AS1 shows linkage to a cold immune microenvironment in COAD. Therefore, we investigated its regulatory mechanism in COAD cells. We performed pathway enrichment analysis of PAN3-AS1 in COAD and showed that PAN3-AS1 might be engaged in the humoral immune response. WFDC13 was one of the humoral immune response-related genes. Now the studies on WFDC13 are also scarce. Coincidentally, in pancreatic cancer, Cao et al. also constructed a risk model using differentially methylated genes, including WFDC13 [40]. Additionally, WFDC13 was found to be a fusion gene in ovarian carcinoma [41]. Interestingly, the results of our *in vitro* experiments confirmed the decreased WFDC13 expression levels in COAD cells after PAN3-AS1 knockdown. Furthermore, in line with the role of PAN3-AS1, WFDC13 expression was also correlated with a cold immune microenvironment, and WFDC13 could also directly inhibit CD8^+^ T cell activity in COAD. Therefore, we hypothesize that PAN3-AS1 might form an inhibitory immune microenvironment by regulating WFDC13 expression in COAD. Cao et al. demonstrated that PAN3-AS1 was an angiogenesis-related lncRNA in pancreatic adenocarcinoma [15]. As is known, angiogenesis is related to immunosuppression. Therefore, our study provides another explanation for the role of PAN3-AS1 in immune escape.

Previous studies have demonstrated that nuclear lncRNAs predominantly function in modulating transcriptional processes, whereas cytosolic lncRNAs modulate RNA decay or the translational process mainly through the ceRNA mechanism. The CeRNA mechanism means that lncRNAs function as endogenous RNAs to compete for binding to microRNAs (miRNAs) to influence downstream target expression [42]. Our study revealed that PAN3-AS1 was primarily localized in the cytoplasm. Based on the online database prediction, we discovered that miR-423-5p could target the PAN3-AS1 RNA and WFDC13 mRNA 3^′^UTR simultaneously. We report for the first time that PAN3-AS1 facilitates WFDC13 expression by competing with miR-423-5p for binding sites. Recently, it has been shown that lncRNA OGFRP1 mediates the formation of CTCF by means of miR-423-5p, which in turn promotes colorectal carcinogenesis, metastasis, and angiogenesis [43]. These results further verified the antitumor role of miR-423-5p. Finally, we also characterized the upstream modulatory mechanism of PAN3-AS1 overexpression in COAD. Enhancers play important regulatory roles in many cancers [44,45]. H3K27ac modification is the optimal biomarker for identifying enhancers. Unexpectedly, we verified the presence of enhancers at the PAN3-AS1 gene locus and elucidated the enhancer-dependent regulation of PAN3-AS1 by *in vitro* experiments. Collectively, epigenetic alteration is an important factor for PAN3-AS1 overexpression in tumors.

### 4.4 Therapeutic Implications

In recent years, RNA-based therapies have been developed, including the use of RNA as drugs and small molecules that target RNA [46]. These therapies have expanded the range of therapeutic targets, providing theoretical support for the subsequent design of drugs targeting PAN3-AS1 in COAD. Importantly, our study screened a series of small molecular drugs to target WFDC13 and validated the effect of three drugs on COAD cell viability. Linsitinib, trametinib, and PD0325901 have been used for clinical trials in many tumor types [47–49]. Therefore, in the future, it could be explored whether combining these drugs with immune checkpoint inhibitors could result in better results for tumor immunotherapy. Of course, our paper has several limitations. For instance, how does WFDC13 regulate the tumor microenvironment? The underlying regulatory network needs further elucidation. And the efficacy and safety of our screened drugs still require additional experimental validation. Does targeting the PAN3-AS1/WFDC13 axis enhance the immunotherapy effect in COAD? This hypothesis needs extra *in vitro* and *in vivo* experiments to substantiate.

## 5 Conclusion

PAN3-AS1 is a valuable biomarker for COAD’s diagnosis and prognosis. PAN3-AS1 expression is linked to low TME and might be a promising candidate for an immunotherapy predictive biomarker. Enhancer-mediated overexpression of PAN3-AS1 promotes WFDC13 expression in COAD. Targeting the PAN3-AS1/miR-423-5p/WFDC13 axis might offer a new therapeutic strategy for improving the immunotherapy efficiency in COAD. Our study highlights a possibility to overcome resistance to immunotherapy by applying PAN3-AS1/WFDC13 inhibitors.

## Data Availability

All the data used in this study are from public databases.

## Abbreviations

**Abbreviations**: **Full** **Name**
ACC: Adrenocortical carcinoma
BLCA: Bladder urothelial carcinoma
BRCA: Breast invasive carcinoma
CESC: Cervical squamous cell carcinoma and endocervical adenocarcinoma
ChIP: Chromatin immunoprecipitation
CHOL: Cholangiocarcinoma
COAD: Colon adenocarcinoma
CRC: Colorectal cancer
CTL: Cytotoxic T cell
DFI: Disease-free interval
DLBC: Diffuse large B cell lymphoma
NSCLC: Non-small cell lung cancer
DSS: Disease-specific survival
FISH: Fluorescence *in situ* hybridization
ESCA: Esophageal carcinoma
GBM: Glioblastoma multiforme
GO: Gene ontology
ICIs: Immune checkpoint inhibitors
IICs: Immune-infiltrating cells
IPS: Immunophenoscore
HRD: Homologous recombination deficiency
HNSC: Head and neck squamous cell carcinoma
KEGG: Kyoto encyclopedia of genes and genomes
KICH: Kidney chromophobe
KIPAN: Pan-kidney cohort (KICH+KIRC+KIRP)
KIRC: Kidney renal clear cell carcinoma
KIRP: Kidney renal papillary cell carcinoma
LAML: Acute myeloid leukemia
LGG: Lower grade glioma
LIHC: Liver hepatocellular carcinoma
LOH: Loss of heterozygosity
LUAD: Lung adenocarcinoma
LUSC: Lung squamous cell carcinoma
MATH: Mutant-allele tumor heterogeneity
MESO: Mesothelioma
MSI: Microsatellite instability
NEO: Neoantigens
OV: Ovarian serous cystadenocarcinoma
OS: Overall survival
PAAD: Pancreatic adenocarcinoma
PCPG: Pheochromocytoma and paraganglioma
PD1: Programmed cell death 1
PDCD1LG2: Programmed cell death 1 ligand 2
PD-L1: Programmed cell death 1 ligand 1
PFI: Progression-free interval
PFS: Progression-free survival
PRAD: Prostate adenocarcinoma
READ: Rectum adenocarcinoma
RIP: RNA immunoprecipitation
SARC: Sarcoma
SKCM: Skin cutaneous melanoma
STAD: Stomach adenocarcinoma
STES: Stomach and esophageal carcinoma
TGCT: Testicular germ cell tumors
THCA: Thyroid carcinoma
THYM: Thymoma
TIDE: Tumor immune dysfunction and exclusion
TMB: Tumor mutational burden
TIME: Tumor immune microenvironment
UCS: Uterine carcinosarcoma
UTR: Untranslated region
UVM: Uveal melanoma

## Appendix A

**Table A1:** Compounds for WFDC13 and their docking energy

Protein	Compounds	Binding energy
WFDC13	linsitinib	−8.18 kcal/mol
WFDC13	trametinib	−5.07 kcal/mol
WFDC13	PD0325901	−2.18 kcal/mol

## References

[1] Siegel RL, Miller KD, Wagle NS, Jemal A. Cancer statistics, 2023. CA Cancer J Clin. 2023;73(1):17–48. doi:10.3322/caac.21763; 36633525

[2] Vasan N, Baselga J, Hyman DM. A view on drug resistance in cancer. Nature. 2019;575(7782):299–309. doi:10.1038/s41586-019-1730-1; 31723286 PMC8008476

[3] Herrmann J. Adverse cardiac effects of cancer therapies: cardiotoxicity and arrhythmia. Nat Rev Cardiol. 2020;17(8):474–502. doi:10.1038/s41569-020-0348-1; 32231332 PMC8782611

[4] Schrock AB, Ouyang C, Sandhu J, Sokol E, Jin D, Ross JS, et al. Tumor mutational burden is predictive of response to immune checkpoint inhibitors in MSI-high metastatic colorectal cancer. Ann Oncol. 2019;30(7):1096–103. doi:10.1093/annonc/mdz134; 31038663

[5] Wang J, Xiu J, Farrell A, Baca Y, Arai H, Battaglin F, et al. Mutational analysis of microsatellite-stable gastrointestinal cancer with high tumour mutational burden: a retrospective cohort study. Lancet Oncol. 2023;24(2):151–61. doi:10.1016/S1470-2045(22)00783-5; 36681091 PMC10599647

[6] Dermani FK, Samadi P, Rahmani G, Kohlan AK, Najafi R. PD-1/PD-L1 immune checkpoint: potential target for cancer therapy. J Cell Physiol. 2019;234(2):1313–25. doi:10.1002/jcp.27172; 30191996

[7] Grote P, Boon RA. LncRNAs coming of age. Circ Res. 2018;123(5):535–7. doi:10.1161/circresaha.118.313447; 30355139

[8] Kafida M, Karela M, Giakountis A. RNA-independent regulatory functions of lncRNA in complex disease. Cancers. 2024;16(15):2728. doi:10.3390/cancers16152728; 39123456 PMC11311644

[9] Dharini AG, Kejamurthy P, Ramya Devi KT. Co-regulation of miRNA and lncRNA on immunosuppression gene: unveiling the regulatory networks in cancer. Nucleosides Nucleotides Nucleic Acids. 2025;6:1–30. doi:10.1080/15257770.2025.2514129; 40479646

[10] Ren Z, Xu Y, Wang X, Ren M. KCNQ1OT1 affects cell proliferation, invasion, and migration through a miR-34a/Notch3 axis in breast cancer. Environ Sci Pollut Res Int. 2022;29(19):28480–94. doi:10.1007/s11356-021-18434-x; 34993814

[11] Liu Y, Kong X, Yu X, Qiao J, Yang K, Li Z, et al. Hepatitis B virus core/capsid protein induces hepatocellular carcinoma progression via long non-coding RNA KCNQ1OT1/miR-335-5p/CDC7 axis. Transl Cancer Res. 2025;14(6):3319–35. doi:10.21037/tcr-2025-233; 40687256 PMC12268880

[12] Wang X, Ren Z, Xu Y, Gao X, Huang H, Zhu F. KCNQ1OT1 sponges miR-34a to promote malignant progression of malignant melanoma via upregulation of the STAT3/PD-L1 axis. Environ Toxicol. 2023;38(2):368–80. doi:10.1002/tox.23687; 36399467

[13] Mostaghimi Y, Haddadi M, Hojjati Z. lncRNA PAN3-AS1 modulates Cilium Assemble signaling pathway through regulation of RPGR as a potential MS diagnostic biomarker: integrated systems biology investigation. J Mol Neurosci. 2025;75(2):49. doi:10.1007/s12031-025-02331-w; 40227518

[14] Ping H, Jia X, Ke H. A novel ferroptosis-related lncRNAs signature predicts clinical prognosis and is associated with immune landscape in pancreatic cancer. Front Genet. 2022;13:786689. doi:10.3389/fgene.2022.786689; 35330729 PMC8940287

[15] Cao G, Chang Y, Yang G, Jiang Y, Han K. A novel risk score model based on four angiogenesis long non-coding RNAs for prognosis evaluation of pancreatic adenocarcinoma. Aging. 2022;14(22):9090–102. doi:10.18632/aging.204387; 36384673 PMC9740371

[16] Shen W, Song Z, Zhong X, Huang M, Shen D, Gao P, et al. Sangerbox: a comprehensive, interaction-friendly clinical bioinformatics analysis platform. Imeta. 2022;1(3):e36. doi:10.1002/imt2.36; 38868713 PMC10989974

[17] Chandrashekar DS, Karthikeyan SK, Korla PK, Patel H, Shovon AR, Athar M, et al. UALCAN: an update to the integrated cancer data analysis platform. Neoplasia. 2022;25(1):18–27. doi:10.1016/j.neo.2022.01.001; 35078134 PMC8788199

[18] Sun D, Wang J, Han Y, Dong X, Ge J, Zheng R, et al. TISCH: a comprehensive web resource enabling interactive single-cell transcriptome visualization of tumor microenvironment. Nucleic Acids Res. 2021;49(1):D1420–30. doi:10.1093/nar/gkaa1020; 33179754 PMC7778907

[19] Tang Z, Kang B, Li C, Chen T, Zhang Z. GEPIA2: an enhanced web server for large-scale expression profiling and interactive analysis. Nucleic Acids Res. 2019;47(1):W556–60. doi:10.1093/nar/gkz430; 31114875 PMC6602440

[20] Gao Y, Shang S, Guo S, Li X, Zhou H, Liu H, et al. Lnc2Cancer 3.0: an updated resource for experimentally supported lncRNA/circRNA cancer associations and web tools based on RNA-seq and scRNA-seq data. Nucleic Acids Res. 2021;49(D1):D1251–8. doi:10.1093/nar/gkaa1006; 33219685 PMC7779028

[21] Jiang P, Gu S, Pan D, Fu J, Sahu A, Hu X, et al. Signatures of T cell dysfunction and exclusion predict cancer immunotherapy response. Nat Med. 2018;24(10):1550–8. doi:10.1038/s41591-018-0136-1; 30127393 PMC6487502

[22] Wang C, Liu H, Yang M, Bai Y, Ren H, Zou Y, et al. RNA-seq based transcriptome analysis of endothelial differentiation of bone marrow mesenchymal stem cells. Eur J Vasc Endovasc Surg. 2020;59(5):834–42. doi:10.1016/j.ejvs.2019.11.003; 31874808

[23] Sunakawa Y, Stintzing S, Cao S, Heinemann V, Cremolini C, Falcone A, et al. Variations in genes regulating tumor-associated macrophages (TAMs) to predict outcomes of bevacizumab-based treatment in patients with metastatic colorectal cancer: results from TRIBE and FIRE3 trials. Ann Oncol. 2015;26(12):2450–6. doi:10.1093/annonc/mdv474; 26416897 PMC4658546

[24] Lei Y, Tang R, Xu J, Wang W, Zhang B, Liu J, et al. Applications of single-cell sequencing in cancer research: progress and perspectives. J Hematol Oncol. 2021;14(1):91. doi:10.1186/s13045-021-01105-2; 34108022 PMC8190846

[25] Morad G, Helmink BA, Sharma P, Wargo JA. Hallmarks of response, resistance, and toxicity to immune checkpoint blockade. Cell. 2021;184(21):5309–37. doi:10.1016/j.cell.2021.09.020; 34624224 PMC8767569

[26] Chan TA, Yarchoan M, Jaffee E, Swanton C, Quezada SA, Stenzinger A, et al. Development of tumor mutation burden as an immunotherapy biomarker: utility for the oncology clinic. Ann Oncol. 2019;30(1):44–56. doi:10.1093/annonc/mdy495; 30395155 PMC6336005

[27] Weng J, Li S, Zhu Z, Liu Q, Zhang R, Yang Y, et al. Exploring immunotherapy in colorectal cancer. J Hematol Oncol. 2022;15(1):95. doi:10.1186/s13045-022-01294-4; 35842707 PMC9288068

[28] Zhang Y, Pang S, Sun B, Zhang M, Jiao X, Lai L, et al. ELOVLs predict distinct prognosis value and immunotherapy efficacy in patients with hepatocellular carcinoma. Front Oncol. 2022;12:884066. doi:10.3389/fonc.2022.884066; 35912257 PMC9334671

[29] Jia Q, Wang A, Yuan Y, Zhu B, Long H. Heterogeneity of the tumor immune microenvironment and its clinical relevance. Exp Hematol Oncol. 2022;11(1):24. doi:10.1186/s40164-022-00277-y; 35461288 PMC9034473

[30] Liu J, Ling Y, Su N, Li Y, Tian S, Hou B, et al. A novel immune checkpoint-related gene signature for predicting overall survival and immune status in triple-negative breast cancer. Transl Cancer Res. 2022;11(1):181–92. doi:10.21037/tcr-21-1455; 35261895 PMC8841573

[31] Yao RW, Wang Y, Chen LL. Cellular functions of long noncoding RNAs. Nat Cell Biol. 2019;21(5):542–51. doi:10.1038/s41556-019-0311-8; 31048766

[32] Wang W, Tang YA, Xiao Q, Lee WC, Cheng B, Niu Z, et al. Stromal induction of BRD4 phosphorylation results in chromatin remodeling and BET inhibitor resistance in colorectal cancer. Nat Commun. 2021;12(1):4441. doi:10.1038/s41467-021-24687-4; 34290255 PMC8295257

[33] Yasukawa Y, Hattori N, Iida N, Takeshima H, Maeda M, Kiyono T, et al. SAA1 is upregulated in gastric cancer-associated fibroblasts possibly by its enhancer activation. Carcinogenesis. 2021;42(2):180–9. doi:10.1093/carcin/bgaa131; 33284950

[34] Narita T, Ito S, Higashijima Y, Chu WK, Neumann K, Walter J, et al. Enhancers are activated by p300/CBP activity-dependent PIC assembly, RNAPII recruitment, and pause release. Mol Cell. 2021;81(10):2166–82. doi:10.1016/j.molcel.2021.03.008; 33765415

[35] Szeto GL, Finley SD. Integrative approaches to cancer immunotherapy. Trends Cancer. 2019;5(7):400–10. doi:10.1016/j.trecan.2019.05.010; 31311655 PMC7467854

[36] Huang AC, Zappasodi R. A decade of checkpoint blockade immunotherapy in melanoma: understanding the molecular basis for immune sensitivity and resistance. Nat Immunol. 2022;23(5):660–70. doi:10.1038/s41590-022-01141-1; 35241833 PMC9106900

[37] Zhang Y, Zhang Z. The history and advances in cancer immunotherapy: understanding the characteristics of tumor-infiltrating immune cells and their therapeutic implications. Cell Mol Immunol. 2020;17(8):807–21. doi:10.1038/s41423-020-0488-6; 32612154 PMC7395159

[38] Rizzo A, Ricci AD, Brandi G. PD-L1, TMB, MSI, and other predictors of response to immune checkpoint inhibitors in biliary tract cancer. Cancers. 2021;13(3):558. doi:10.3390/cancers13030558; 33535621 PMC7867133

[39] Janjigian YY, Sanchez-Vega F, Jonsson P, Chatila WK, Hechtman JF, Ku GY, et al. Genetic predictors of response to systemic therapy in esophagogastric cancer. Cancer Discov. 2018;8(1):49–58. doi:10.1158/2159-8290.CD-17-0787; 29122777 PMC5813492

[40] Cao T, Wu H, Ji T. Bioinformatics-based construction of prognosis-related methylation prediction model for pancreatic cancer patients and its application value. Front Pharmacol. 2023;14:1086309. doi:10.3389/fphar.2023.1086309; 36969862 PMC10034005

[41] Smebye ML, Agostini A, Johannessen B, Thorsen J, Davidson B, Tropé CG, et al. Involvement of *DPP9* in gene fusions in serous ovarian carcinoma. BMC Cancer. 2017;17(1):642. doi:10.1186/s12885-017-3625-6; 28893231 PMC5594496

[42] Venkatesh J, Wasson MD, Brown JM, Fernando W, Marcato P. LncRNA-miRNA axes in breast cancer: novel points of interaction for strategic attack. Cancer Lett. 2021;509:81–8. doi:10.1016/j.canlet.2021.04.002; 33848519

[43] Dong H, Liu Q, Chen C, Lu T, Xu K. LncRNA OGFRP1 promotes angiogenesis and epithelial-mesenchymal transition in colorectal cancer cells through miR-423-5p/CTCF axis. Immunobiology. 2022;227(2):152176. doi:10.1016/j.imbio.2022.152176; 35066433

[44] Ye B, Fan D, Xiong W, Li M, Yuan J, Jiang Q, et al. Oncogenic enhancers drive esophageal squamous cell carcinogenesis and metastasis. Nat Commun. 2021;12(1):4457. doi:10.1038/s41467-021-24813-2; 34294701 PMC8298514

[45] Li Q, Lv X, Han C, Kong Y, Dai Z, Huo D, et al. Enhancer reprogramming promotes the activation of cancer-associated fibroblasts and breast cancer metastasis. Theranostics. 2022;12(17):7491–508. doi:10.7150/thno.75853; 36438487 PMC9691365

[46] Yu AM, Choi YH, Tu MJ. RNA drugs and RNA targets for small molecules: principles, progress, and challenges. Pharmacol Rev. 2020;72(4):862–98. doi:10.1124/pr.120.019554; 32929000 PMC7495341

[47] Davis SL, Eckhardt SG, Diamond JR, Messersmith WA, Dasari A, Weekes CD, et al. A phase I dose-escalation study of linsitinib (OSI-906), a small-molecule dual insulin-like growth factor-1 receptor/insulin receptor kinase inhibitor, in combination with irinotecan in patients with advanced cancer. Oncologist. 2018;23(12):1409–e140. doi:10.1634/theoncologist.2018-0315; 30139840 PMC6292546

[48] Prasath V, Boutrid H, Wesolowski R, Abdel-Rasoul M, Timmers C, Lustberg M, et al. Phase II study of MEK inhibitor trametinib alone and in combination with AKT inhibitor GSK2141795/uprosertib in patients with metastatic triple negative breast cancer. Breast Cancer Res Treat. 2025;210(1):179–89. doi:10.1007/s10549-024-07551-z; 39644403 PMC12796985

[49] van Geel RMJM, van Brummelen EMJ, Eskens FALM, Huijberts SCFA, de Vos FYFL, Lolkema MPJK, et al. Phase 1 study of the pan-HER inhibitor dacomitinib plus the MEK1/2 inhibitor PD-0325901 in patients with KRAS-mutation-positive colorectal, non-small-cell lung and pancreatic cancer. Br J Cancer. 2020;122(8):1166–74. doi:10.1038/s41416-020-0776-z; 32147669 PMC7156736

